# SARS-CoV-2 Receptor ACE2 Is an Interferon-Stimulated Gene in Human Airway Epithelial Cells and Is Detected in Specific Cell Subsets across Tissues

**DOI:** 10.1016/j.cell.2020.04.035

**Published:** 2020-05-28

**Authors:** Carly G.K. Ziegler, Samuel J. Allon, Sarah K. Nyquist, Ian M. Mbano, Vincent N. Miao, Constantine N. Tzouanas, Yuming Cao, Ashraf S. Yousif, Julia Bals, Blake M. Hauser, Jared Feldman, Christoph Muus, Marc H. Wadsworth, Samuel W. Kazer, Travis K. Hughes, Benjamin Doran, G. James Gatter, Marko Vukovic, Faith Taliaferro, Benjamin E. Mead, Zhiru Guo, Jennifer P. Wang, Delphine Gras, Magali Plaisant, Meshal Ansari, Ilias Angelidis, Heiko Adler, Jennifer M.S. Sucre, Chase J. Taylor, Brian Lin, Avinash Waghray, Vanessa Mitsialis, Daniel F. Dwyer, Kathleen M. Buchheit, Joshua A. Boyce, Nora A. Barrett, Tanya M. Laidlaw, Shaina L. Carroll, Lucrezia Colonna, Victor Tkachev, Christopher W. Peterson, Alison Yu, Hengqi Betty Zheng, Hannah P. Gideon, Caylin G. Winchell, Philana Ling Lin, Colin D. Bingle, Scott B. Snapper, Jonathan A. Kropski, Fabian J. Theis, Herbert B. Schiller, Laure-Emmanuelle Zaragosi, Pascal Barbry, Alasdair Leslie, Hans-Peter Kiem, JoAnne L. Flynn, Sarah M. Fortune, Bonnie Berger, Robert W. Finberg, Leslie S. Kean, Manuel Garber, Aaron G. Schmidt, Daniel Lingwood, Alex K. Shalek, Jose Ordovas-Montanes, HCA Lung Biological Network, Nicholas Banovich, Pascal Barbry, Alvis Brazma, Tushar Desai, Thu Elizabeth Duong, Oliver Eickelberg, Christine Falk, Michael Farzan, Ian Glass, Muzlifah Haniffa, Peter Horvath, Deborah Hung, Naftali Kaminski, Mark Krasnow, Jonathan A. Kropski, Malte Kuhnemund, Robert Lafyatis, Haeock Lee, Sylvie Leroy, Sten Linnarson, Joakim Lundeberg, Kerstin Meyer, Alexander Misharin, Martijn Nawijn, Marko Z. Nikolic, Jose Ordovas-Montanes, Dana Pe’er, Joseph Powell, Stephen Quake, Jay Rajagopal, Purushothama Rao Tata, Emma L. Rawlins, Aviv Regev, Paul A. Reyfman, Mauricio Rojas, Orit Rosen, Kourosh Saeb-Parsy, Christos Samakovlis, Herbert Schiller, Joachim L. Schultze, Max A. Seibold, Alex K. Shalek, Douglas Shepherd, Jason Spence, Avrum Spira, Xin Sun, Sarah Teichmann, Fabian Theis, Alexander Tsankov, Maarten van den Berge, Michael von Papen, Jeffrey Whitsett, Ramnik Xavier, Yan Xu, Laure-Emmanuelle Zaragosi, Kun Zhang

**Affiliations:** 1 Program in Health Sciences & Technology, Harvard Medical School & Massachusetts Institute of Technology, Boston, MA 02115, USA; 2 Institute for Medical Engineering & Science, Massachusetts Institute of Technology, Cambridge, MA 02139, USA; 3 Koch Institute for Integrative Cancer Research, Massachusetts Institute of Technology, Cambridge, MA 02139, USA; 4 Ragon Institute of MGH, MIT, and Harvard, Cambridge, MA 02139, USA; 5 Broad Institute of MIT and Harvard, Cambridge, MA 02142, USA; 6 Harvard Graduate Program in Biophysics, Harvard University, Cambridge, MA 02138, USA; 7 Department of Chemistry, Massachusetts Institute of Technology, Cambridge, MA 02139, USA; 8 Program in Computational & Systems Biology, Massachusetts Institute of Technology, Cambridge, MA 02139, USA; 9 Computer Science & Artificial Intelligence Lab, Massachusetts Institute of Technology, Cambridge, MA 02139, USA; 10 Africa Health Research Institute, Durban, South Africa; 11 School of Laboratory Medicine and Medical Sciences, College of Health Sciences, University of KwaZulu-Natal, Durban, South Africa; 12 University of Massachusetts Medical School, Worcester, MA 01655, USA; 13 Department of Microbiology, Harvard Medical School, Boston, MA 02115, USA; 14 Program in Virology, Harvard Medical School, Boston, MA 02115, USA; 15 John A. Paulson School of Engineering & Applied Sciences, Harvard University, Cambridge, MA 02138, USA; 16 Program in Immunology, Harvard Medical School, Boston, MA 02115, USA; 17 Division of Pediatric Hematology/Oncology, Boston Children’s Hospital, Boston, MA 02115, USA; 18 Division of Gastroenterology, Hepatology, and Nutrition, Boston Children’s Hospital, Boston, MA 02115, USA; 19 Aix-Marseille University, INSERM, INRA, C2VN, Marseille, France; 20 Université Côte d’Azur, CNRS, IPMC, Sophia-Antipolis, France; 21 Comprehensive Pneumology Center & Institute of Lung Biology and Disease, Helmholtz Zentrum München, Munich, Germany; 22 German Center for Lung Research, Munich, Germany; 23 Institute of Computational Biology, Helmholtz Zentrum München, Munich, Germany; 24 Research Unit Lung Repair and Regeneration, Helmholtz Zentrum München, Munich, Germany; 25 Division of Neonatology, Department of Pediatrics, Vanderbilt University Medical Center, Nashville, TN 37232, USA; 26 Divison of Allergy, Pulmonary, and Critical Care Medicine, Department of Medicine, Vanderbilt University Medical Center, Nashville, TN 37232, USA; 27 Center for Regenerative Medicine, Massachusetts General Hospital, Boston, MA 02114, USA; 28 Division of Gastroenterology, Brigham and Women’s Hospital, Boston, MA 02115, USA; 29 Division of Allergy and Clinical Immunology, Department of Medicine, Brigham and Women’s Hospital, Boston, MA 02115, USA; 30 University of California, Berkeley, CA 94720, USA; 31 University of Washington, Seattle, WA 98195, USA; 32 Dana Farber Cancer Institute, Boston, MA 02115, USA; 33 Harvard Medical School, Boston, MA 02115, USA; 34 Stem Cell & Gene Therapy Program, Fred Hutchinson Cancer Research Center, Seattle, WA 98109, USA; 35 Department of Medicine, University of Washington, Seattle, WA 98195, USA; 36 Division of Gastroenterology and Hepatology, Seattle Children’s Hospital, Seattle, WA 98145, USA; 37 Department of Microbiology & Molecular Genetics, University of Pittsburgh School of Medicine, Pittsburgh, PA 15219, USA; 38 Center for Vaccine Research, University of Pittsburgh School of Medicine, Pittsburgh, PA 15261, USA; 39 Division of Pulmonary, Allergy, and Critical Care Medicine, University of Pittsburgh School of Medicine, Pittsburgh, PA 15213, USA; 40 UPMC Children’s Hospital of Pittsburgh, Pittsburgh, PA 15224, USA; 41 Department of Pediatrics, University of Pittsburgh School of Medicine, Pittsburgh, PA 15224, USA; 42 Department of Infection, Immunity & Cardiovascular Disease, The Medical School and The Florey Institute for Host Pathogen Interactions, University of Sheffield, Sheffield, S10 2TN, UK; 43 Department of Medicine, Vanderbilt University Medical Center, Nashville, TN 37232, USA; 44 Department of Cell and Developmental Biology, Vanderbilt University Medical Center, Nashville, TN 37240, USA; 45 Department of Veterans Affairs Medical Center, Nashville, TN 37212, USA; 46 Department of Infection & Immunity, University College London, London, UK; 47 Harvard T.H. Chan School of Public Health, Boston, MA 02115, USA; 48 Department of Mathematics, Massachusetts Institute of Technology, Cambridge, MA 02139, USA; 49 Harvard Stem Cell Institute, Cambridge, MA 02138, USA

**Keywords:** scRNA-seq, interferon, ISG, ACE2, SARS-CoV-2, COVID-19, influenza, non-human primate, human, mouse

## Abstract

There is pressing urgency to understand the pathogenesis of the severe acute respiratory syndrome coronavirus clade 2 (SARS-CoV-2), which causes the disease COVID-19. SARS-CoV-2 spike (S) protein binds angiotensin-converting enzyme 2 (ACE2), and in concert with host proteases, principally transmembrane serine protease 2 (TMPRSS2), promotes cellular entry. The cell subsets targeted by SARS-CoV-2 in host tissues and the factors that regulate *ACE2* expression remain unknown. Here, we leverage human, non-human primate, and mouse single-cell RNA-sequencing (scRNA-seq) datasets across health and disease to uncover putative targets of SARS-CoV-2 among tissue-resident cell subsets. We identify *ACE2* and *TMPRSS2* co-expressing cells within lung type II pneumocytes, ileal absorptive enterocytes, and nasal goblet secretory cells. Strikingly, we discovered that *ACE2* is a human interferon-stimulated gene (ISG) *in vitro* using airway epithelial cells and extend our findings to *in vivo* viral infections. Our data suggest that SARS-CoV-2 could exploit species-specific interferon-driven upregulation of *ACE2*, a tissue-protective mediator during lung injury, to enhance infection.

## Highlights

• Meta-analysis of human, non-human primate, and mouse single-cell RNA-seq datasets for putative SARS-CoV-2 targets
• Type II pneumocytes, nasal secretory cells, and absorptive enterocytes are *ACE2*^+^*TMPRSS2*^+^
• Interferon and influenza increase *ACE2* in human nasal epithelia and lung tissue
• Mouse *Ace2* is not upregulated by interferon, raising implications for disease modeling

## Graphical Abstract

**Figure:**
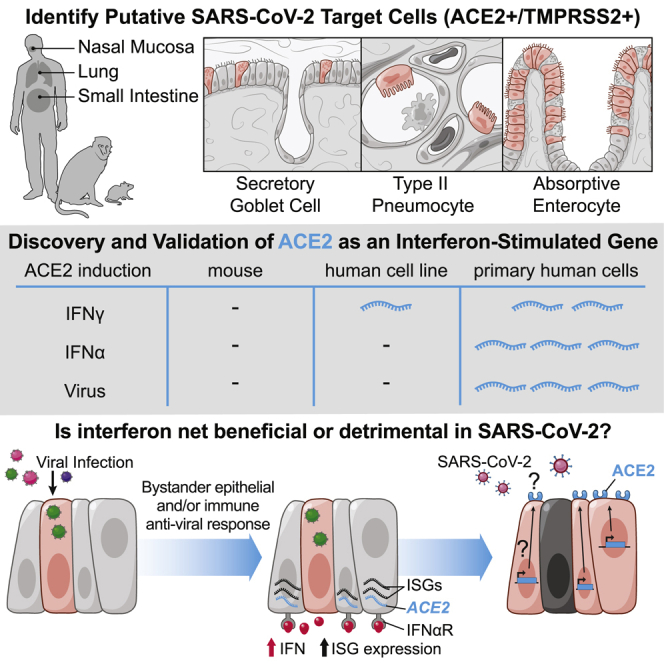

## Teaser

Analysis of single-cell RNA-seq datasets from human, non-human primate, and mouse barrier tissues identifies putative cellular targets of SARS-CoV-2 on the basis of *ACE2* and *TMPRSS2* expression. *ACE2* represents a previously unappreciated interferon-stimulated gene in human, but not mouse, epithelial tissues, identifying anti-viral induction of a host tissue-protective mechanism, but also a potential means for viral exploitation of the host response.

## Introduction

Human coronaviruses (CoVs) are single-stranded positive-sense RNA viruses that can cause mild to severe respiratory disease (Fung and Liu, 2019). Over the past two decades, zoonotic transmission events have led to the emergence of two highly pathogenic CoVs: severe acute respiratory syndrome (SARS)-CoV and Middle East respiratory syndrome (MERS)-CoV. SARS-CoV-2, which causes the disease known as COVID-19, was first reported in late 2019 (Coronaviridae Study Group of the International Committee on Taxonomy of V., 2020, Lu et al., 2020, Paules et al., 2020). COVID-19 is characterized by pneumonia, fever, cough, and occasional diarrhea (Guan et al., 2020, Holshue et al., 2020, Huang et al., 2020), and SARS-CoV-2 RNA has been reliably detected in nasopharyngeal swabs, sputum, and stool samples (Wang et al., 2020, Wölfel et al., 2020, Zou et al., 2020). As of April 19, 2020, SARS-CoV-2 continues to spread worldwide, and there are over 2,401,379 confirmed cases, 165,044 deaths, and 623,903 recovered individuals in 185 countries and regions (Dong et al., 2020a). Early models of COVID-19 transmission dynamics estimate one infectious individual infects slightly over two individuals; travel restrictions reduce that spread to one individual, although these figures might evolve as more accurate epidemiological data become available (Kucharski et al., 2020).

Work during the first SARS-CoV epidemic identified the human host factor angiotensin-converting enzyme 2 (ACE2) as the receptor for SARS-CoV (Li et al., 2003). SARS-CoV-2 spike (S) protein has been experimentally shown to bind ACE2 on host cells with significantly higher affinity than SARS-CoV-S (Hoffmann et al., 2020, Wrapp et al., 2020). The main host protease that mediates S protein activation on primary target cells and initial viral entry is the type II transmembrane serine protease TMPRSS2 (Glowacka et al., 2011, Hoffmann et al., 2020, Iwata-Yoshikawa et al., 2019, Matsuyama et al., 2010, Shulla et al., 2011, Walls et al., 2020). Other host proteases, such as furin, have also been suggested to promote the pathogenesis of this pandemic SARS-CoV-2 clade, but when and where they process S protein remains to be determined (Böttcher-Friebertshäuser et al., 2013, Bugge et al., 2009, Coutard et al., 2020, Walls et al., 2020). Binding of SARS-CoV-S to ACE2 results in receptor-mediated internalization (Grove and Marsh, 2011, Kuba et al., 2005). Importantly, ACE2 functions as a key tissue-protective component during severe acute lung injury (Imai et al., 2005, Kuba et al., 2005).

A tissue-level basis for understanding SARS-CoV tropism was proposed based on ACE2 histological staining and expression in human epithelia of the lung and small intestine (Hamming et al., 2004, Harmer et al., 2002, Jonsdottir and Dijkman, 2016). However, unlike the specific expression of CDHR3 (the rhinovirus-C receptor), which is resolved to ciliated epithelial cells of the upper airway (Griggs et al., 2017), the specific cell subsets within each tissue that express *ACE2* remain unknown. Identifying the cell subsets targeted by SARS-CoV-2 (ACE2^+^) and those at greatest risk of direct infection (ACE2^+^TMPRSS2^+^) is critical for understanding and modulating host defense mechanisms and viral pathogenesis.

After cellular detection of viral entry into a host cell, interferon (IFN) induction of interferon-stimulated genes (ISGs) is essential for host antiviral defense in mice, non-human primates (NHPs), and humans (Bailey et al., 2014, Deeks et al., 2017, Dupuis et al., 2003, Everitt et al., 2012, Schneider et al., 2014, Utay and Douek, 2016). There are three distinct types of IFNs: type I IFNs (IFN-α and IFN-β), type II IFNs (IFN-γ), and type III IFNs (IFN-λ) (Broggi et al., 2020, Müller et al., 1994, Stetson and Medzhitov, 2006). Each appears to converge on almost indistinguishable responses, mediated through the binding of STAT1 homodimers or STAT1/STAT2 heterodimers to ISGs. However, mounting evidence suggests that each type of IFN might have a non-redundant role in host defense or immunopathology, particularly at epithelial barriers (Broggi et al., 2020, Iwasaki et al., 2017, Iwasaki and Pillai, 2014, Jewell et al., 2010).

Although the host response to SARS-CoV highlighted a role for IFNs, most studies assessed the effect of IFN restriction in cell lines that might not fully recapitulate the repertoire of ISGs present in primary human target cells (Bailey et al., 2014, de Lang et al., 2006, Sainz et al., 2004, Zheng et al., 2004). One study of SARS-CoV suggested the timing of the type I IFN response was critical *in vivo* (Channappanavar et al., 2016). Clinical therapy using approved IFNs has been attempted for SARS-CoV, MERS-CoV, and SARS-CoV-2 in the absence of a controlled trial to mixed effect, resulting in anecdotal evidence suggesting either rapid improvement or worsening of symptoms (Dong et al., 2020b, Lei et al., 2020, Li and De Clercq, 2020). Elucidating tissue- and cell-type-specific ISGs and their activity is essential for understanding the role of IFNs in host defense during human SARS-CoV-2 infection.

Massively parallel single-cell RNA-sequencing (scRNA-seq) is transforming our ability to comprehensively map the cell types, subsets, and states present during health and disease in barrier tissues (Ordovas-Montanes et al., 2020, Ordovas-Montanes et al., 2018, Smillie et al., 2019). This has been particularly evident in the elucidation of novel human epithelial and stromal cell subsets and states (Ordovas-Montanes et al., 2018, Regev et al., 2017, Ruiz García et al., 2019, Schiller et al., 2019, Smillie et al., 2019, Vieira Braga et al., 2019). Recently, scRNA-seq has been applied to better understand the cellular variation present during viral infection *in vitro* and *in vivo* (Russell et al., 2018, Steuerman et al., 2018). Global single-cell profiling efforts such as the Human Cell Atlas (HCA) initiative are ideally poised to rapidly share critical data and enhance our understanding of disease during emergent public health challenges (Sungnak et al., 2020).

Here, using published and unpublished datasets (all from non-SARS-CoV-2-infected samples), we analyze human, NHP, and mouse tissues that have been clinically identified to harbor virus in patients exhibiting COVID-19 symptoms. We provide a cautionary note on the interpretation of the scRNA-seq data presented below, given that many factors such as dissociation, profiling method, and sequencing depth can influence results (STAR Methods). Here, we focus our analysis and discussion on the specific subsets where *ACE2* and *TMPRSS2* are enriched and on relative comparisons *within* each dataset, rather than *between* datasets or equivalence to absolute numbers of total cells. Across several studies of human and NHP tissues, we found ISGs upregulated in *ACE2*-expressing cells.

Strikingly, by treating primary human upper airway basal cells with distinct types of inflammatory cytokines, we demonstrate that IFN-α drives *ACE2* expression. Human influenza infection also induces broader expression of *ACE2* in upper airway epithelial cells and is corroborated by publicly available databases. Overall, our data provide motivation to better understand the trade-offs of antiviral and/or IFN therapy in humans infected with SARS-CoV-2 in order to balance host restriction, tissue tolerance, and viral enhancement mechanisms (Davidson et al., 2015, Fung and Liu, 2019, Imai et al., 2005, Iwasaki et al., 2017, Kuba et al., 2005, Lei et al., 2020, Medzhitov et al., 2012, Zou et al., 2014). Importantly, although our findings identify similar cell subsets enriched for *Ace2* in mice, neither *in vitro* nor *in vivo* IFN-stimulation nor *in vivo* viral challenge substantially alter *Ace2* expression levels. The dynamic, species-specific and multifaceted role of IFN raises implications for pre-clinical COVID-19 disease modeling.

## Results

### Lung Epithelial Cell Expression of Host Factors Used by SARS-CoV-2 in Non-Human Primates and Humans

**Figure 1:**
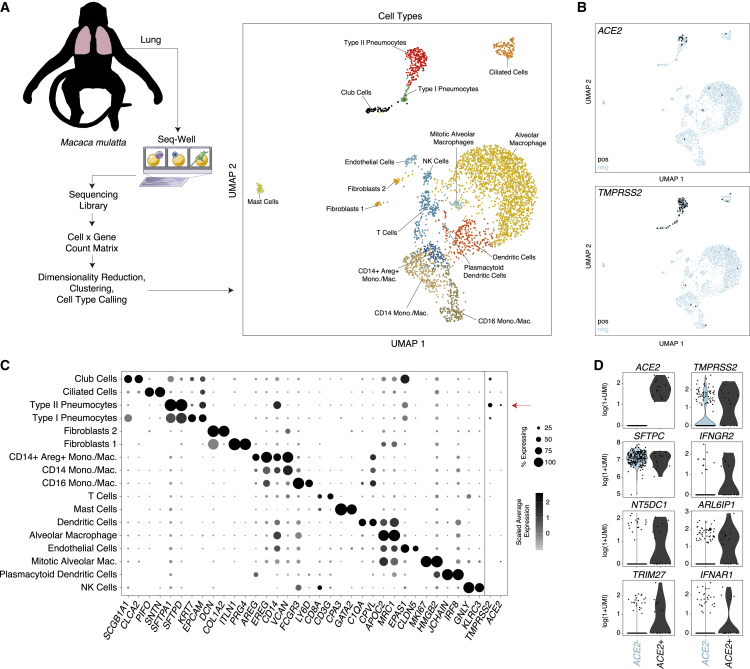
Expression of *ACE2* in Type II Pneumocytes in Healthy Lungs of Non-human Primates (A) Schematic of protocol for isolation of lung tissue at necropsy from healthy non-human primates (*M. mulatta*, n = 3), creation of scRNA-seq libraries by using Seq-Well v1, and computational analysis to identify cell types by using unbiased methods. UMAP projection of 3,793 single cells, points colored by cell identity (see STAR Methods). (B) Uniform manifold approximation and projection (UMAP) as in (A), points colored by detection of *ACE2* (coronavirus receptor, top) or *TMPRSS2* (coronavirus S protein priming for entry, bottom). Color coding is as follows: black, RNA positive; blue, RNA negative. (C) Dot plot of 2 defining genes for each cell type (Table S1) (Bonferroni-adjusted p < 0.001) and *ACE2* and *TMPRSS2*. Dot size represents fraction of cells within that type expressing a given gene, and color intensity represents binned count-based expression amount (log(scaled UMI+1)) among expressing cells. *ACE2* is enriched in type II pneumocytes (6.7% expressing, Bonferroni-adjusted p = 8.62E−33), as is *TMPRSS2* (29.5% expressing, Bonferroni-adjusted p = 8.73E−153). Of all type II pneumocytes, 3.8% co-express *ACE2* and *TMPRSS2* (Table S9). Red arrow indicates cell type with largest proportion of *ACE2*^+^*TMPRSS2*^+^ cells. (D) Genes differentially expressed among *ACE2*^+^ and *ACE2*^−^ type II pneumocytes. (SCDE package, FDR-adjusted p < 0.05 for *IFNGR2*, *NT5DC1*, *ARL6IP1*, and *TRIM27*; full results can be found in Table S1). See also Table S1.

To investigate which cells within human and NHP tissues represent likely SARS-CoV-2 targets, we analyzed new and existing scRNA-seq datasets to assess which cell types express *ACE2*, alone or with *TMPRSS2*. In a previously unpublished dataset consisting of NHP (*Macaca mulatta*) lung tissue collected after necropsy of healthy adult animals and analyzed by using Seq-Well v1 (Gierahn et al., 2017), we recovered at least 17 distinct major cell types, including various lymphoid, myeloid, and stromal populations (Figures 1A–1C; Table S1; STAR Methods). *ACE2* and *TMPRSS2* were primarily expressed in epithelial cells, with 6.7% of type II pneumocytes expressing *ACE2* and 3.8% co-expressing *ACE2* and *TMPRSS2* (Figures 1B and 1C). Notably, the only double-positive cells observed were classified within the type II pneumocyte population; however, we also identified *TMPRSS2* expression within club cells, ciliated epithelial cells, and type I pneumocytes, albeit at diminished abundance and frequency compared with type II pneumocytes (Figure 1C; Table S1).

Next, we compared *ACE2*^+^ with *ACE2*^−^ type II pneumocytes to explore broader gene programs that differentiate putative SARS-CoV-2 target cells from cells of a similar phenotype and ontogeny (Figure 1D; Table S1). Among genes significantly upregulated in *ACE2*^+^ type II pneumocytes, we observed *IFNGR2* (false discovery rate [FDR]-adjusted p = 0.022), a receptor for type II IFNs. Notably, previous work has demonstrated limited anti-viral potency of IFN-γ for SARS-associated coronaviruses, compared with that of type I IFNs, at least *in vitro* (Sainz et al., 2004, Zheng et al., 2004). Other co-regulated genes of potential interest include *TRIM27* (FDR-adjusted p = 0.025), as well as *NT5DC1* (FDR-adjusted p = 0.003) and *ARL6IP1* (FDR-adjusted p = 0.047), which were upregulated in the A549 adenocarcinoma alveolar basal epithelial cell line after exposure to IFN-α and IFN-γ for 6 h (Sanda et al., 2006). We found *IFNAR1* consistently expressed among both *ACE2*^+^ type II pneumocytes and *ACE2*^+^*TMPRSS2*^+^ co-expressing type II pneumocytes, but its level of upregulation compared with all remaining pneumocytes did not meet statistical significance (FDR-adjusted p = 0.11). This analysis finds *ACE2*^+^ cells enriched within a rare fraction of secretory cells in NHPs and that *ACE2* expression is co-regulated with genes involved in IFN responses.

**Figure 2:**
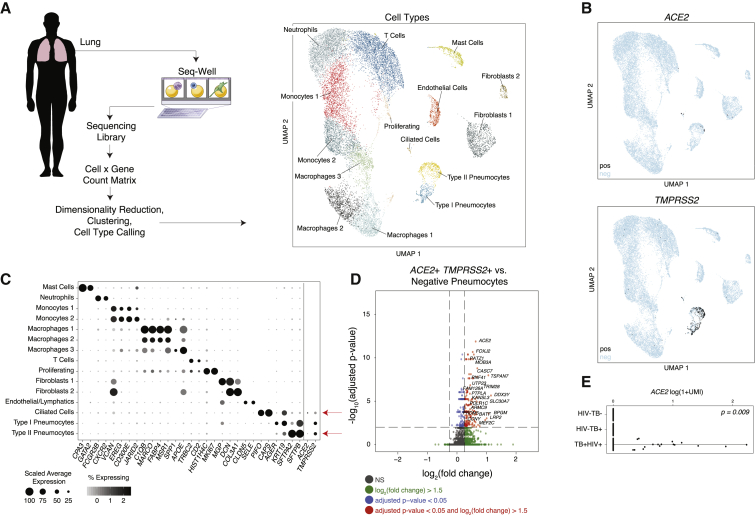
Select Lung Epithelial Cells from Control, HIV-1-Infected, and *Mycobacterium*-*tuberculosis*-Infected Human Donors Co-Express *ACE2* and *TMPRSS2* (A) Schematic of protocol for isolation of human lung tissue from surgical excess, creation of scRNA-seq libraries by using Seq-Well S^3^, and computational analysis to identify cell types by using unbiased methods. Shown on the right is a UMAP projection of 18,915 cells across 8 donors (n = 3 TB^+^HIV^+^; n = 3 TB^+^; n = 2 non-infected patients). Cells represented by points, colored according to cell type (see STAR Methods). (B) UMAP projection as in (A), points colored by detection of *ACE2* (top) or *TMPRSS2* (bottom). Color coding is as follows: black, RNA positive; blue, RNA negative. (C) Dot plot of 2 defining genes for each cell type (FDR-adjusted p < 0.001), and *ACE2* and *TMPRSS2*; dot size represents fraction of cells within cell type expressing a given gene, and color intensity represents binned count-based expression amount (log(scaled UMI+1)) among expressing cells. All cluster-defining genes are provided in Table S2. Red arrow indicates cell types with largest proportion of *ACE2*^+^*TMPRSS2*^+^ cells. (D) Volcano plot identifying significantly upregulated genes in *ACE2*^+^*TMPRSS2*^+^ pneumocytes compared with all remaining pneumocytes. Red points represent genes with a FDR-adjusted p < 0.05, and log_2_(fold change) >1.5. Text highlighting specific genes; the full list is available in Table S2. (E) Expression of *ACE2* across human donors by HIV and TB status (p = 0.009 by likelihood-ratio test). See also Table S2.

To assess whether the findings from NHP lung cells were similarly present in humans, we analyzed a previously unpublished scRNA-seq dataset derived from surgical resections of fibrotic lung tissue collected with Seq-Well S^3^ (Hughes et al., 2019). Unsupervised analysis identified multiple cell types and subtypes of immune cells (Figures 2A–2C; STAR Methods), as defined by the genes displayed in Figure 2C (full lists available in Table S2). Here, we found that *ACE2* and *TMPRSS2* were primarily expressed within type II pneumocytes and ciliated cells, in line with our analysis of the NHP-derived cells (Figures 1 and 2A, 2B). In type II pneumocytes (identified by unique expression of surfactant proteins *SFTPC*, *SFTPB*, and *SFTPA1*), we found 1.4% of cells expressing *ACE2* (FDR-adjusted p = 1.35E−21), 34.2% expressing *TMPRSS2* (FDR-adjusted p < 1E−300), and 0.8% co-expressing both. In ciliated cells, we found 7% were *ACE2*^+^ (FDR-adjusted p = 5E−64), 24.6% were *TMPRSS2*^+^ (FDR-adjusted p = 3.8E−30), and 5.3% co-expressed both.

As above, to assess for cellular pathways significantly co-expressed within putative target cells for SARS-CoV-2, we computed differentially expressed genes between *ACE2*^+^*TMPRSS2*^+^ type II pneumocytes and all other type II pneumocytes (Figures 2C and 2D; Table S2). We found significant enrichment of *BATF* among *ACE2*^+^*TMPRSS2*^+^ cells (FDR-adjusted p = 3.25E−7), which has been demonstrated previously to be upregulated by type I and type II IFNs (Murphy et al., 2013). Of note, we also observed *TRIM28* co-expressed with *ACE2* and *TMPRSS2* among type II pneumocytes in this dataset (FDR-adjusted p = 2.34E−9), which might play a role in potentiating an IFN response in lung epithelial cells (Krischuns et al., 2018). Within this cohort of donors, 3 individuals were human immunodeficiency virus (HIV)^+^ and diagnosed with active tuberculosis, 3 donors had active tuberculosis and were HIV^−^, and 2 were negative for both pathogens. Surprisingly, we found that all of the *ACE2*^+^ cells across all cell types were derived from HIV^+^ *Mycobacterium tuberculosis* (Mtb)^+^ donors despite approximately equivalent recovery of epithelial cell types from all donors (likelihood-ratio test, p = 0.009) (Figure 2E). Given limited cell and patient numbers combined with potential sampling biases, we caution that this observation requires much broader cohorts to validate a potential role for co-infections; still, we note our observation is suggestive of a role for chronic IFNs in the induction of *ACE2*, given that HIV infection is associated with persistent upregulation of ISGs, and we observed elevated amounts of *IFNAR2*, *IFI30*, and *IKBKB* (Utay and Douek, 2016) (FDR-adjusted p = 1.1E−6, 8.8E−9, 1.57E−7, respectively; HIV^+^ versus HIV^−^ epithelial cells).

**Figure S1:**
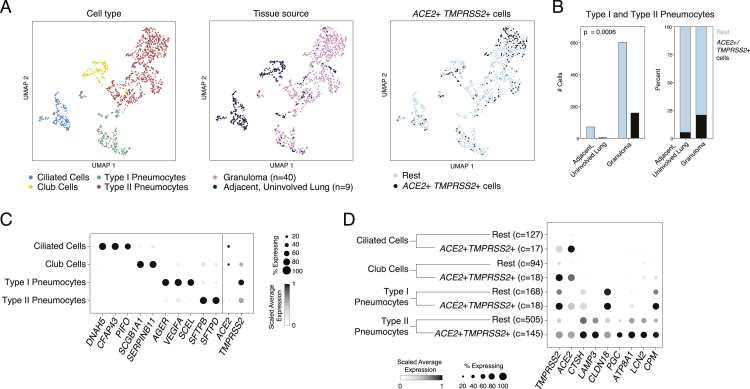
NHP Tuberculosis Infected Lung and Granuloma, Related to Figures 1 and 2 (A). UMAP projection of epithelial cells (1,099 cells) colored by annotated cell type, tissue source, and gating as *ACE2*^+^*TMPRSS2*^+^ cells. *ACE2*^+^*TMPRSS2*^+^ cells comprise 11% of ciliated cells, 16% of club cells, 10% type I pneumocytes, and 22% type II pneumocytes. Data generated using Seq-Well S^3^ (Table S3). (B). Number of cells (left) and % (right) *ACE2*^+^*TMPRSS2*^+^ cells by tissue source (granuloma versus uninvolved lung) and cell type. Ciliated cells and club cells were omitted from this analysis as we detected too few cells (< 7 total cells) belonging to these clusters in the granulomas. Statistical significance assessed by Fisher Exact Test (Table S3). (C). Dot plot of top cluster defining genes for each epithelial cell type and *ACE2* and *TMPRSS2*. Dot size represents fraction of cells expressing, and color intensity represents average log(normalized UMI + 1) among all cells in each group scaled between 0 and 1 by gene. *ACE2* expression is enriched in club cells (Bimodal test, Bonferroni-corrected p < 0.001), ciliated cells (p < 0.005), and type I pneumocytes (p < 0.001). *TMPRSS2* expression is enriched in type I pneumocytes (p < 0.001) and ciliated cells (p < 0.001) (Table S3). (D). Dot plot of genes differentially expressed between *ACE2^+^TMPRSS2*^*+*^ epithelial cells versus rest (Bimodal test, Bonferroni-corrected p < 0.01, log fold change > 0.5). (Table S3, c = number of cells, n = number of animals).

Next, using a previously unpublished scRNA-seq dataset consisting of granuloma and adjacent, uninvolved lung samples from Mtb-infected NHPs (*Macaca fascicularis*) collected with Seq-Well S^3^, we identified subsets of epithelial cells expressing *ACE2* and *TMPRSS2* (Figure S1; Table S3; STAR Methods). The majority of *ACE2*^+^*TMPRSS2*^+^ cells were, once again, type II pneumocytes (22%) and type I pneumocytes (9.7%) and were largely enriched within granulomatous regions compared with those in adjacent uninvolved lung (Figures S1B and S1C) (p = 0.006, Fisher Exact Test). *ACE2*^+^*TMPRSS2*^+^ type II pneumocytes expressed significantly higher amounts of antimicrobial effectors such as *LCN2* compared with remaining type II pneumocytes (Figure S1D). Cells with club cell/secretory, type I pneumocyte, and ciliated cell types also contained some *ACE2*^+^*TMPRSS2*^+^ cells, but we did not have sufficient power to detect significantly differentially expressed genes between these cells and other cells within those clusters. Altogether, we identify *ACE2*^+^*TMPRSS2*^+^ cells in lower airways of humans and NHPs with consistent cellular phenotypes and evidence supporting a potential role for IFN-associated inflammation in upregulation of *ACE2*.

### Ileal Absorptive Enterocytes Express Host Factors Used by SARS-CoV-2

**Figure 3:**
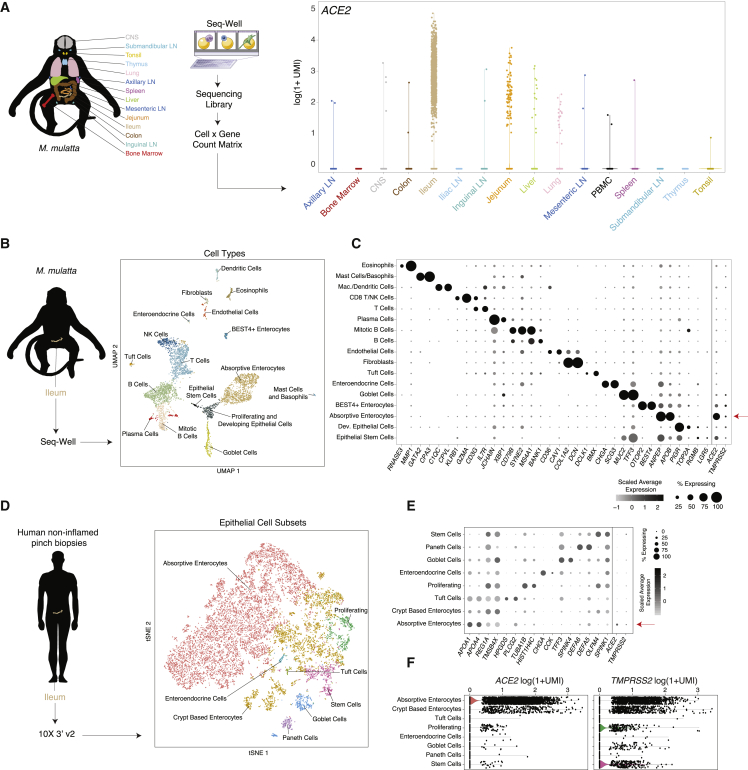
NHP and Human Ileal Absorptive Enterocytes Co-Express *ACE2* and *TMPRSS2* (A) Expression *ACE2* across diverse tissues in healthy NHPs (n = 3 animals; 52,858 cells). (B) Schematic of protocol for isolation of NHP ileum (n = 5) at necropsy for scRNA-seq using Seq-Well v1, and computational pipeline to identify cell types by using unbiased methods. Shown on the right is a UMAP projection of 4,515 cells colored by cell type. (C) Dot plot of 2 defining genes for each cell type, with *ACE2* and *TMPRSS2*. Dot size represents fraction of cells within cell type expressing a given gene, and color intensity represents binned count-based expression amounts (log(scaled UMI+1)) among expressing cells. All cluster defining genes are provided in Table S4. Red arrow indicates cell type with largest proportion of *ACE2*^+^*TMPRSS2*^+^ cells. (D) Schematic of protocol for isolation of human ileal cells from endoscopic pinch biopsies in non-inflamed regions (n = 13). Shown on the right is a tSNE plot of 13,689 epithelial cells selected from original dataset generated by 10x 3′ v2 (see Figure S2), colored by cellular subsets. (E). Dot plot of 2 defining genes for each cell type, with *ACE2* and *TMPRSS2*. Dot size represents fraction of cells within cell type expressing a given gene, and color intensity represents binned count-based expression amounts (log(scaled UMI+1)) among expressing cells. All cluster defining genes are provided in Table S5. Red arrow indicates cell type with largest proportion of *ACE2*^+^*TMPRSS2*^+^ cells. (F). Expression of *ACE2* (left) and *TMPRSS2* (right) among all epithelial subsets from human donors. See also Figure S2 and Tables S4 and S5.

Next, we examined several other tissues for *ACE2*-expressing cells on the basis of the location of hallmark symptoms of COVID-19, focusing on the gastrointestinal tract due to reports of clinical symptoms and viral shedding (Xiao et al., 2020). Leveraging a previously unpublished scRNA-seq atlas of NHP *(M. mulatta)* tissues collected with Seq-Well v1, we observed that the majority of *ACE2*^+^ cells reside in the small intestine, principally within the ileum, jejunum, and, to a lesser extent, the liver and colon (Figure 3A; STAR Methods). Critically, we note that, in this experiment, the dissociation method used on each tissue was optimized to preserve immune cell recovery, and therefore under-sampled stromal and epithelial populations, as well as neurons from the brain. Within the ileum, we identified *ACE2*^+^ cells as absorptive enterocytes on the basis of specific expression of *ACE2* within cells defined by *APOA1*, *SI*, *FABP6*, and *ENPEP,* among others, by a likelihood-ratio test (Figures 3B and 3C) (p < 1E−300, 62% of all absorptive enterocytes; see Table S4). All other epithelial subtypes expressed *ACE2* to a lesser extent, and variably co-expressed *ACE2* with *TMPRSS2* (see Table S4 for full statistics).

**Figure S2:**
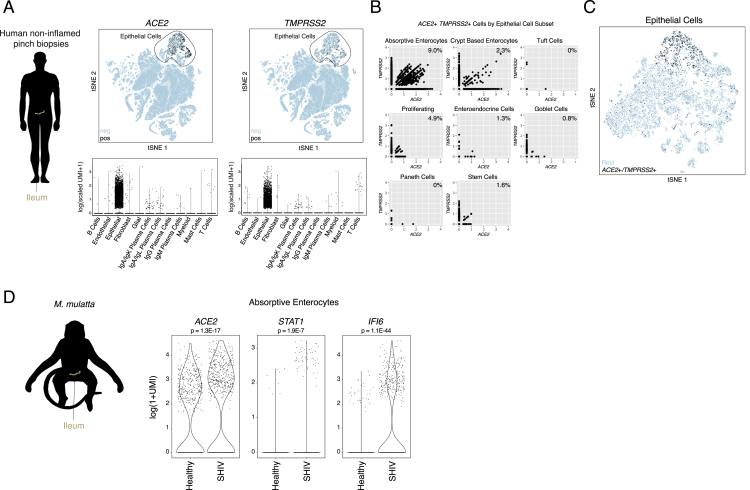
Human and NHP Ileum, Related to Figure 3 (A). Top: tSNE projection of all cells from healthy pediatric human ileum within a previously-unpublished 10x 3′ v2 dataset (115,569 cells). Black: higher expression of *ACE2* (left), *TMPRSS2* (right). Bottom: Corresponding violin plots of expression values for *ACE2* (left) and *TMPRSS2* (right). Solid line: epithelial cells. (B). Co-expression of *ACE2* and *TMPRSS2* by epithelial cell subset. Number indicates % of *ACE2*^+^*TMPRSS2*^+^ cells by cell subset. (C). tSNE projection of 13,689 cells as in Figure 3D, cells colored by co-expression of *ACE2* and *TMPRSS2* (black). (D). Expression of *ACE2* and canonical interferon-responsive genes among absorptive enterocytes from Healthy (n = 2) and SHIV-infected, anti-retroviral treated animals (n = 3). Bonferroni-adjusted p-values by Wilcoxon test (healthy: 510 cells, SHIV-infected: 636 cells).

Persistent viral RNA in rectal swabs has been detected in pediatric infection, even after negative nasopharyngeal tests (Xu et al., 2020). In an additional dataset consisting of endoscopic biopsies from the terminal ileum of a human pediatric cohort (n = 13 donors, ranging in age from 10 to 18 years old), collected with 10X 3′ v2, we confirmed a large abundance of *ACE2*^+^ cells with selective expression within absorptive enterocytes (29.7% *ACE2*^+^, FDR-adjusted p = 2.46E−100) (Figures 3D and 3E; Table S5; STAR Methods). Furthermore, we identified a subset (888 cells, ∼6.5% of all epithelial cells) that co-express both genes (Figures S2A–S2C). We performed differential expression testing and GO-term enrichment using these cells relative to matched non-expressers to highlight putative biological functions enriched within them, such as metabolic processes and catalytic activity, and to identify shared phenotypes of *ACE2*^+^*TMPRSS2*^+^ ileal cells across both human and NHP cohorts (Table S5). We speculate that viral targeting of these cells, taken from patients without overt clinical viral infection, might help explain intestinal symptoms. Finally, we compared ileal absorptive enterocytes from healthy NHPs and NHPs infected with simian-human immunodeficiency virus (SHIV) and then treated for 6 months with anti-retroviral therapy (animal and infection characteristics published in Colonna et al., 2018) (STAR Methods). We found significant upregulation of *ACE2*, *STAT1*, and *IFI6* within the absorptive enterocytes of SHIV-infected animals (which maintain chronically elevated amounts of IFNs and ISGs) compared with those of uninfected controls (FDR-adjusted p < 2E-7) (Figure S2D) (Deeks et al., 2017, Utay and Douek, 2016).

### Upper Airway Expression of Host Factors Used by SARS-CoV-2

**Figure 4:**
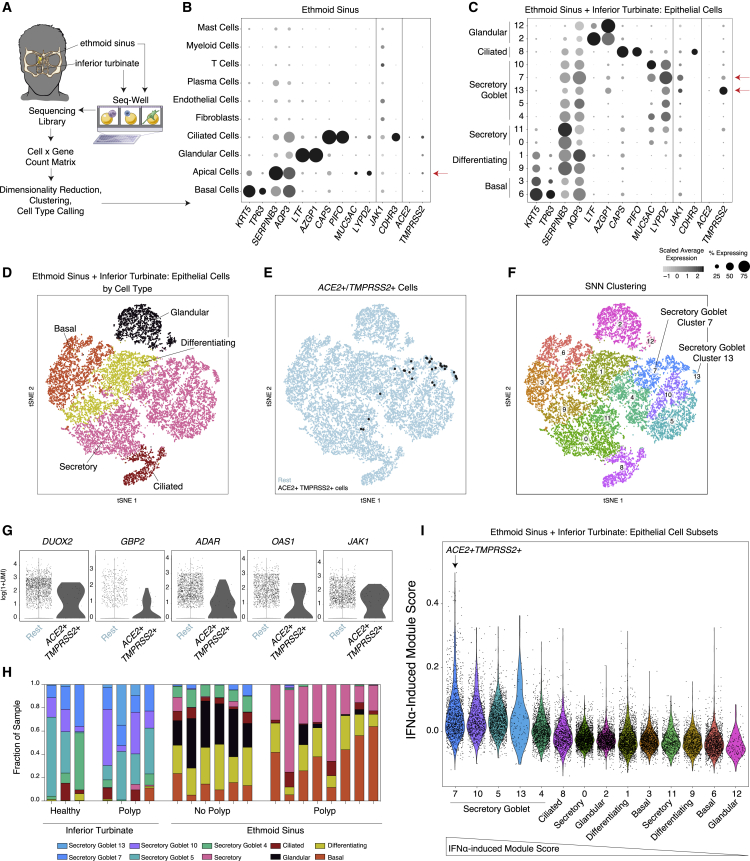
Healthy and Allergic Inflamed Human Nasal Mucosa Co-Express *ACE2* and *TMPRSS2* in a Subset of Goblet Secretory Cells (A) Schematic for sampling of n = 12 ethmoid sinus surgical samples and n = 9 inferior turbinate nasal scrapings to generate scRNA-seq libraries by using Seq-Well v1. See Ordovas-Montanes et al., (2018). (B) Dot plot of all cell types from ethmoid-sinus-derived cells (n = 6 non-polyp CRS samples, n = 6 polyp CRS samples). Two defining genes for each cell type, in addition to *CDHR3* (rhinovirus receptor), *ACE2*, *TMPRSS2*, and *JAK1*. Dot size represents fraction of cells within that type expressing a given gene, and color intensity represents binned count-based expression amounts (log(scaled UMI+1)) among expressing cells (see Table S6 for statistics by subset). Red arrow indicates cell types with largest proportion of *ACE2*^+^*TMPRSS2*^+^ cells. (C) Dot plot for 2 defining genes for each cell type identified from granular clustering of epithelial cells (18,325 single cells) derived from both ethmoid sinus and inferior turbinate sampling (healthy inferior turbinate [3,681 cells; n = 3 samples], polyp-bearing patient inferior turbinate [1,370 cells; n = 4 samples], non-polyp ethmoid sinus surgical samples [5,928 cells; n = 6 samples], and polyp surgical and scraping samples directly from polyp in ethmoid sinus [7,346 cells; n = 8 samples]). Red arrow indicates cell type with largest proportion of *ACE2*^+^*TMPRSS2*^+^ cells. (D) tSNE of 18,325 single epithelial cells from inferior turbinate and ethmoid sinus (omitting immune cells). Colored by cell types 3,152 basal, 3,089 differentiating, 8,840 secretory, 1,105 ciliated, and 2,139 glandular cells. (E) tSNE as in (D), identifying epithelial cells co-expressing *ACE2* and *TMPRSS2* (30 cells, black points). (F) tSNE as in (D), colored by detailed cell types with higher granularity, as in (C). (G) Individual differentially expressed genes between *ACE2*^+^*TMPRSS2*^+^ cells and all other secretory epithelial cells (see Table S6 for full gene list with statistics). Bonferroni-adjusted likelihood-ratio test p < 0.02 for all genes displayed. (H) Stacked bar plot of each subset of epithelial cells among all epithelial cells by donor (each bar) and sampling location (noted below graph) (unpaired t test p < 0.00035 for Secretory Goblet 7 inferior turbinate versus ethmoid sinus; see Table S6 for raw values). (I) Violin plot of cell clusters in respiratory epithelial cells (from Figures 4C and 4F) ordered by average expression of IFN-α-induced gene signatures, presented as a gene module score; non-normal distribution by Lilliefors test, Mann-Whitney U-test p = 2.2E−16, 1.21 effect size, IFN-α signature for Secretory Goblet Cluster 7 versus all epithelial cells. Arrow indicates cluster containing majority *ACE2*^+^*TMPRSS2*^+^ cells. See also Figure S3 and Table S6.

To identify potential viral target cells in nasal and sinus tissue, two regions that are frequently primary sites of exposure for coronaviruses, we analyzed existing scRNA-seq datasets from the human upper airway (inferior turbinate and ethmoid sinus mucosa) across a spectrum of healthy donors and individuals with allergic inflammation due to chronic rhinosinusitis (CRS) collected with Seq-Well v1 (Figure 4A; STAR Methods) (Ordovas-Montanes et al., 2018). We had previously noted a significantly enriched IFN-dominated gene signature in inferior turbinate secretory epithelial cells from both healthy and CRS donors compared with CRS samples from the ethmoid sinus, which were significantly enriched for interleukin-4 (IL-4)/IL-13 gene signatures (Giovannini-Chami et al., 2012, Ordovas-Montanes et al., 2018). We speculate that these cells, taken from clinically non-virally infected patients, yet constantly exposed to environmental viruses, might provide one of the earliest locations for coronaviruses to infect before spreading to other tissues. We observed significant enrichment of *ACE2* expression in apical epithelial cells and, to a lesser extent, ciliated cells compared with all cell types recovered from surgically resected mucosa (1% of apical epithelial cells, FDR-adjusted p = 4.55E−6, n.s. in ciliated cells) (Figure 4B; Table S6).

To better map putative SARS-CoV-2 targets among epithelial subsets, we employed a finer-grained clustering method applied to both ethmoid sinus surgical specimens and scrapings from the inferior turbinate and ethmoid sinus (Figures 4C–4F). Once again, we observed selective expression of *ACE2* within a minority of cell types, with 1.3% of all secretory cells expressing *ACE2* (Figure 4C) (FDR-adjusted p = 0.00023), specifically sub-clusters 7 and 13, which represent two varieties of secretory epithelial cell (Figures 4C, 4F, and 4G). Cluster 7 secretory cells are marked by *S100P*, *LYPD2*, *PSCA*, *CEACAM5*, and *STEAP4*; encompass some *MUC5AC* goblet cells; and contain the most significantly enriched *ACE2* and *TMPRSS2* expression (4% express *ACE2*, FDR-adjusted p = 7.32E−28; 28% express *TMPRSS2*, FDR-adjusted p = 2.15E−132; Table S6). We next explicitly gated cells by their *TMPRSS2* and *ACE2* expression, identifying a rare subset that co-expresses both, the majority of which fall within the “Secretory Cluster 7” cell type (Figures 4E and 4F) (30 cells, ∼0.3% of all upper airway secretory cells, 1.6% of goblet “Secretory Cluster 7”). These findings are aligned with concurrent work by the HCA Lung Biological Network on human nasal scRNA-seq data, which identified nasal secretory cells to be enriched for *ACE2* and *TMPRSS2* expression (Sungnak et al., 2020).

**Figure S3:**
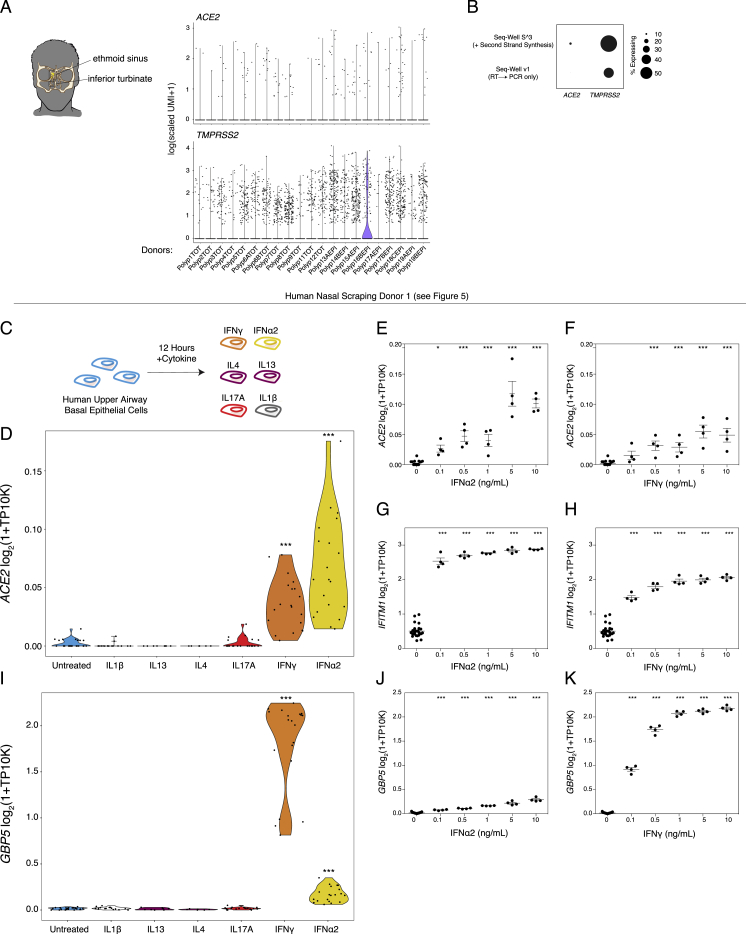
Nasal and Sinus Mucosa, Related to Figures 4 and 5 (A). Expression of *ACE2* and *TMPRSS2* across donors. (B). Enhanced capture of *ACE2* mRNA with second strand synthesis protocol employed in Seq-Well S^3^. Dot size represents fraction of cells expressing. (C). Cultured human primary basal epithelial cells at confluence were treated with increasing doses (0.1 to 10ng/mL) of IFNα2, IFNγ, IL-4, IL-13, IL-17A, and IL-1B for 12 h and bulk RNA-seq analysis was performed (Replicate experiment using Human Donor 1 as in Figure 5) (D). *ACE2* expression by stimulation condition. Wilcoxon test between each cytokine (combined doses) versus rest: IFNα Bonferroni-adjusted p = 4.1E-07; IFNγ Bonferroni-adjusted p = 9.3E-03; all else n.s. ^∗∗∗^ p < 0.001. (E). *ACE2* expression by IFNα2 dose. Bonferroni-corrected t-test compared to 0 ng/mL condition: ^∗∗∗^ p < 0.001, ^∗^ p < 0.05. (F). *ACE2* expression by IFNγ dose. Bonferroni-corrected t-test compared to 0 ng/mL condition: ^∗∗∗^ p < 0.001, ^∗^ p < 0.05. (G). *IFITM1* expression by IFNα2 dose. Bonferroni-corrected t-test compared to 0 ng/mL condition: ^∗∗∗^ p < 0.001. (H). *IFITM1* expression by IFNγ dose. Bonferroni-corrected t-test compared to 0 ng/mL condition: ^∗∗∗^ p < 0.001. (I). *GBP5* expression among cultured human primary basal epithelial cells. Wilcoxon test: IFNα versus IFNγ Bonferroni-adjusted p = 2.94E-07; IFNγ Bonferroni-adjusted p = 9.3E-03. TP10K: transcripts per 10,000 reads. ^∗∗∗^ p < 0.001. (J). *GBP5* expression by IFNα2 dose. Bonferroni-corrected t-test compared to 0 ng/mL condition: ^∗∗∗^ p < 0.001. (K). *GBP5* expression by IFNγ dose. Bonferroni-corrected t-test compared to 0 ng/mL condition: ^∗∗∗^ p < 0.001.

Although we identified co-expression of *ACE2 and TMPRSS2* in few airway cells overall, we detected *ACE2* and *TMPRSS2* single- and double-positive cells in over 20 donors and thus posit that these genes are enriched in secretory cells and are not a product of individual-patient-driven variability (Figure S3A). Inferior turbinate scrapings collected on Seq-Well S^3^, which increases the resolution of lower-abundance transcripts compared with Seq-Well v1, revealed consistent and specific expression restricted to goblet secretory cells, but at a greater detection frequency in samples from the same donors (Figure S3B) (*ACE2*^+^ from 4.7% v1 to 9.8% S^3^; *ACE2*^+^*TMPRSS2*^+^ from 1.9% v1 to 4% S^3^) (Hughes et al., 2019). Using the gated *ACE2*^+^*TMPRSS2*^+^ cells, we tested for differentially expressed genes compared to the remaining secretory epithelial cells (full results provided in Table S6). Notably, we observed significant upregulation of *ADAR*, *GBP2*, *OAS1*, *JAK1*, and *DUOX2* (FDR adjusted, all p < 0.02) within *ACE2*^+^*TMPRSS2*^+^ cells, potentially indicative of IFN signaling (Figure 4G). Almost all “Secretory Cluster 7” cells were from inferior turbinate scrapings of healthy and allergically inflamed individuals, few cells were from the ethmoid sinus tissue of patients with chronic rhinosinusitis without nasal polyps, and no cells were detected in polyp tissue (Figure 4H). Gene Ontology (GO) analysis of enriched genes in double-positive cells include processes related to intracellular cytoskeleton and macromolecular localization and catabolism, potentially involved in viral particle entry, packaging, and exocytosis (Fung and Liu, 2019).

We next utilized IFN-inducible gene sets of relevance to human airway epithelial cells, which we derived from a prior study by performing differential expression on a published dataset where air-liquid interface cultures from primary human nasal epithelial cells were treated with IFN-αA/D, IFN-β1a, IFN-γ, IL-4, or IL-13 (Giovannini-Chami et al., 2012, Ordovas-Montanes et al., 2018). Using these gene lists, we scored the human nasal epithelial cells analyzed by scRNA-seq described in Figures 4C and 4F and found significant concomitant upregulation of the IFN-α-stimulated gene set within *ACE2*^+^*TMPRSS2*^+^ secretory goblet cluster 7 (Figure 4I).

### Type I Interferon IFN-α Drives *ACE2* Expression in Primary Human Nasal Epithelial Cells

The meta-analysis described above consistently identified an association between *ACE2* expression and canonical ISGs or components of the IFN-signaling pathway. This prompted us to investigate whether IFNs might play an active role in regulating *ACE2* expression levels in specific target cell subsets, thus potentially allowing for a tissue-protective host response or increased viral binding of SARS-CoV-2 through ACE2. Our initial literature search indicated that IFN-γ and IL-4 downregulate the SARS-CoV receptor ACE2 in Vero E6 cells (African green monkey kidney epithelial cells [de Lang et al., 2006]), appearing to invalidate this hypothesis. Relatedly, *in vitro* stimulation of A549 cells, a commonly used cell line model for lung epithelia, with IFN-α, IFN-γ, and IFN-α+IFN-γ for 24 h did not identify *ACE2* as an ISG (Russell et al., 2018). This is potentially explained by recent work that aimed to understand SARS-CoV-2 receptor usage by performing screening studies within cell line models and found that A549 cells did not express *ACE2* and therefore represents a poor model to understand regulation of this gene (Letko et al., 2020). While conducting experiments to directly test the hypothesis that *ACE2* is an ISG, we noted in our own gene lists used for scoring from Ordovas-Montanes et al., 2018 and in a supplementary extended table available from Giovannini-Chami et al., 2012 that *ACE2* was in upregulated gene lists after exposure to Type I IFN.

We directly tested whether IFN-α induces *ACE2* in primary human upper airway epithelial cells in greater detail. We cultured human primary basal (stem and progenitors) epithelial cells to confluence and treated them with increasing doses (0.1–10 ng/mL) of IFN-α2, IFN-γ, IL-4, IL-13, IL-17A, or IL-1β for 12 h and then performed bulk RNA-seq (Figure S3C). Only IFN-α2 and IFN-γ led to upregulation of *ACE2* over the time period tested, and compared with all other cytokines, IFN-α2 lead to greater and more significant upregulation over all doses tested (Figure S3D,Wilcoxon test: IFN-α2 FDR-adjusted p = 4.1E−07; IFN-γ p = 9.3E-03,Figures S3E and S3F, all statistical tests compared with 0 ng/mL dose). We confirmed substantial and dose-dependent induction of canonical members of the interferon response after IFN-α2 and IFN-γ (Figures S3G and S3H). Conversely, we found that IFN-γ, relative to IFN-α2, induced potent upregulation of *GBP5*, a GTPase-like protein thought to act as a viral restriction factor through inhibiting furin-mediated protease activity, which could limit viral processing from infected cells, whereas IFN-α2 more robustly induced *IFITM1* (Figure S3G–S3K) (Braun and Sauter, 2019).

**Figure 5:**
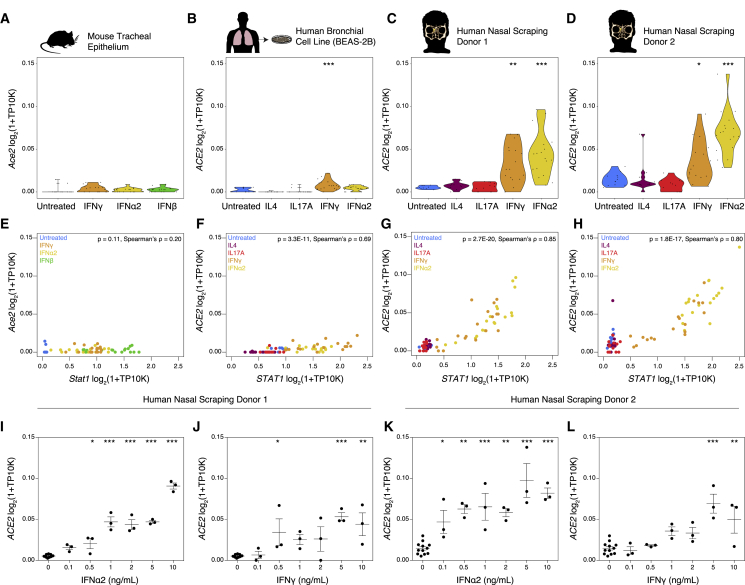
*ACE2* is an Interferon-Stimulated Gene in Primary Human Barrier Tissue Epithelial Cells (A–D) Basal epithelial cells from distinct sources were cultured to confluence and treated with increasing doses (0.1–10 ng/mL) of IFN-α2, IFN-γ, IL-4, IL-17A, and/or IFN-β for 12 h and bulk RNA-seq analysis was performed. Expression of *ACE2* (human) or *Ace2* (mouse) by cell type and stimulation condition. (A) Primary mouse basal cells from tracheal epithelium are shown. (B) BEAS-2B human bronchial cell line is shown. (C) Primary human basal cells from nasal scraping, Donor 1, is shown. (D) Primary human basal cells from nasal scraping, Donor 2. Abbreviation is as follows: TP10K, transcripts per 10,000 reads. ^∗∗∗^p < 0.001, ^∗∗^p < 0.01, ^∗^p < 0.05, Bonferroni-corrected t test compared with untreated condition. (E–H) Co-expression of *STAT1/Stat1* and *ACE2/Ace2* by cell type. (E) Primary mouse basal cells from tracheal epithelium are shown. (F) BEAS-2B human bronchial cell line is shown. (G) Primary human basal cells from nasal scraping, Donor 1, are shown. (H) Primary human basal cells from nasal scraping, Donor 2 are shown. Abbreviation is as follows: TP10K, transcripts per 10,000 reads. Statistical significance assessed by Spearman’s rank correlation. (I–L) Expression of *ACE2* in primary human basal cells from nasal scrapings across a range of concentrations of IFN-γ or IFN-α2. (I) IFN-α2 dose response in Donor 1 (p < 0.001 by one-way ANOVA) is shown. (J) IFN-γ dose response in Donor 1 (p < 0.01 by one-way ANOVA) is shown. (K) IFN-α2 dose response in Donor 2 (p < 0.001 by one-way ANOVA) is shown. (L) IFN-γ dose response in Donor 2 (p < 0.001 by one-way ANOVA). Abbreviation is as follows: TP10K, transcripts per 10,000 reads. ^∗∗∗^p < 0.001, ^∗∗^p < 0.01, ^∗^p < 0.05, Bonferroni-corrected post hoc testing compared with 0 ng/mL condition. See also Figures S3 and S4 and Table S7.

To further extend and substantiate these findings, as above, we stimulated primary mouse tracheal basal cells, the commonly used human bronchial cell line BEAS-2B, and upper airway basal cells from two human donors (Figure 5A-D). We confirmed appropriate induction of an IFN response in each cell type by performing differential expression testing between untreated cells and IFN-treated cells for each condition (Table S7). Within each cell type, stimulation with IFN-α2, IFN-γ, or IFN-β resulted in dose-dependent upregulation of canonical ISGs, including *STAT1/Stat1*, *BST2/Bst2*, *XAF1/Xaf1*, *IFI35/Ifi35*, *MX1/Mx1*, and *GBP2/Gbp2.* Notably, *Ace2* expression was not robustly induced in basal cells derived from healthy mouse trachea under any interferon stimulation condition (Figure 5A). The magnitude of *ACE2* upregulation was diminished in BEAS-2B cells compared to that in our original findings in primary human upper airway epithelial cells, but reached statistical significance compared with that of the untreated condition after IFN-γ exposure (Figure 5B). In primary basal cells derived from healthy nasal mucosa, we confirmed significant induction of *ACE2* after IFN-α2 stimulation and, to a lesser extent, after stimulation with IFN-γ (IFN-α2-stimulated: both Bonferroni-adjusted p < 0.001; IFN-γ-stimulated: both Bonferroni-adjusted p < 0.05) (Figures 5C and 5D). Expression of *ACE2* was significantly correlated with expression of *STAT1* in all human cell types, with a larger effect size and correlation coefficient in primary human basal cells (Figure 5E-H). These experiments support a relationship between induction of the canonical IFN response, including key transcription factors and transcriptional regulation of the *ACE2* locus. Finally, among primary human samples, we confirmed the dose-dependence of *ACE2* upregulation after IFN-α2 or IFN-γ treatment and significant induction of *ACE2* after IFN-α2 stimulation at concentrations as low as 0.1–0.5 ng/mL (Figure 5I-L).

**Figure S4:**
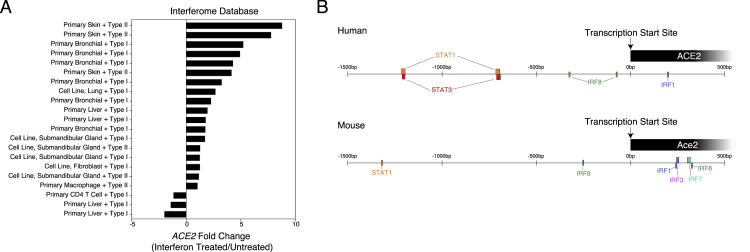
Published Studies of Epithelial Cells Following Interferon Treatment Related to Figure 5 (A). Fold change of *ACE2* expression among human or mouse datasets following Type I or Type II interferon treatment compared to untreated control. Generated from publicly available microarray data curated at interferome.org. Includes all studies with abs(fold-change) > 1. (B). Location of transcription factors binding regions spanning −1500 bp to +500 bp from the transcription start site of *ACE2* (human, top) or *Ace2* (mouse, bottom). Generated from TRANSFAC data using the interferome.org database (Matys et al., 2003, Rusinova et al., 2013).

Next, using a publicly available resource (interferome.org) that hosts genomic and transcriptomic data from cells or tissues treated with IFN, we queried *ACE2* expression within human and mouse cells, searching for datasets with a log_2_-fold-change of >1 or < −1 compared with untreated samples, including all IFN types (Rusinova et al., 2013). We recovered 21 datasets spanning 8 distinct primary tissues or cell lines with non-trivial changes in *ACE2* expression after both type I and type II IFN treatment (Figure S4A). We observed substantial upregulation of *ACE2* in primary skin and primary bronchial cells treated with either type I or type II IFN (> 5-fold upregulation compared with that in untreated cells), in strong support of our *in vitro* data (Figures 5C, 5D, 5G–5L, and S3D–S3F). Immune cell types, such as CD4 T cells and macrophages, were noticeably absent from datasets with a significant change in *ACE2* expression after IFN stimulation or were even found to downregulate *ACE2* (e.g., primary CD4 T cells + type I IFN) (Figure S4A, and in our analysis of scRNA-seq peripheral blood mononuclear cell data from Butler et al., (2018); data not shown).

Given that the majority of cells robustly upregulating *ACE2* were epithelial, this observation potentially explains why previous analyses to define canonical ISGs within immune populations did not identify *ACE2* as an induced gene. Furthermore, using both Transcription Factor database (TRANSFAC) data hosted by the interferome database, as well as chromatin immunoprecipitation sequencing (ChIP-seq) data (provided by the ENCODE Factorbook repository), we found evidence for STAT1, STAT3, IRF8, and IRF1 binding sites within −1500–500 bp of the transcription start site of *ACE2* (all in human studies, Figure S4B) (Gerstein et al., 2012, Matys et al., 2003, Wang et al., 2012, Wang et al., 2013). This finding is supportive of our current hypothesis that *ACE2* represents a previously unappreciated ISG in epithelial cells within barrier tissues.

**Figure S5:**
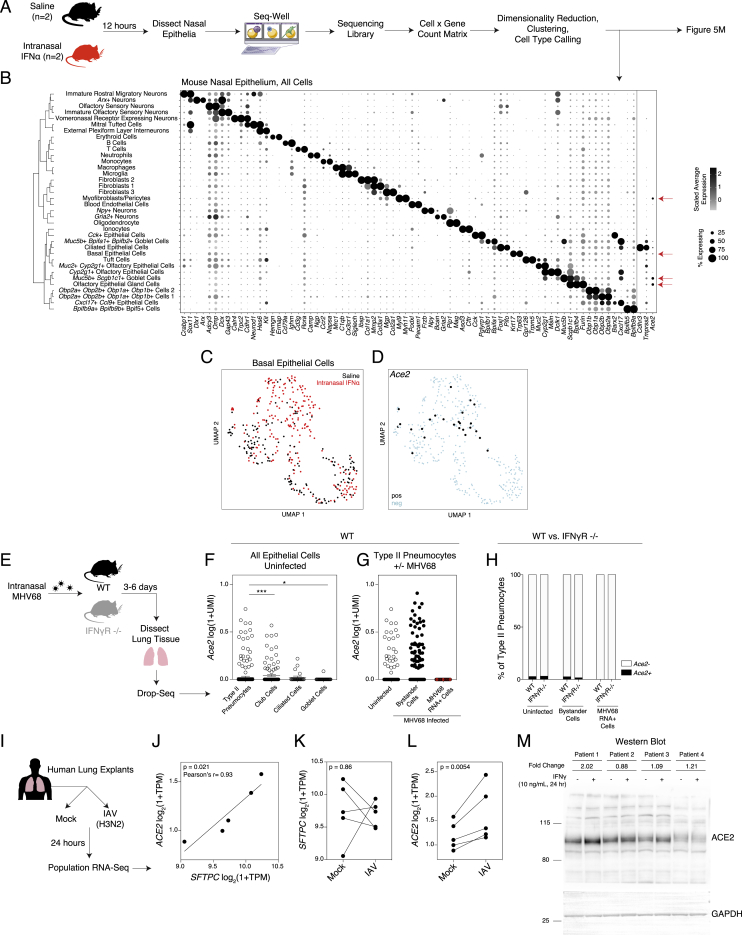
Mouse Nasal Epithelium Following Interferon-α Exposure Related to Figure 6 (A). Schematic: mice were exposed to 10,000 units of IFN-α or saline by intranasal application (n = 2 per group). After 12 h, animals were sacrificed and nasal epithelium was dissected and dissociated for scRNA-seq using Seq-Well S^3^. (B). Dot plot of 2 defining genes for each cell type, with *Ace2*, *Tmprss2*, and *Cdhr3*. Dot size represents fraction of cells within cell type expressing, and color intensity binned count-based expression level (log(scaled UMI+1)) among expressing cells. All cluster defining genes are provided in Table S8. Red arrows: cell types with largest proportion of *Ace2*+ cells. Dendrogram (left) by person correlation over differentially expressed genes with Ward clustering. (C). UMAP of Basal Epithelial Cells (380 cells) across 4 mice. Black: Saline-treated mouse; red: IFN-α treated. (D). UMAP of Basal Epithelial Cells as in C, points colored by detection of *Ace2*. Black: RNA positive, blue: RNA negative (6.6% *Ace2*^+^, Bonferroni-adjusted p = 1.1E-10 for Basal Epithelial Cell expression versus all other cells). (E). Schematic: wildtype (WT) and IFNγ-receptor knockout (IFNγR−/−) mice were infected intranasally with murine gamma-herpesvirus-68 (MHV68). Cells from whole lung were digested for scRNA-seq using Drop-seq (yielding 5,558 Epcam+ cells). (F). Expression of *Ace2* by epithelial cell type, wild type (WT) mice. Statistical significance by Wilcoxon rank sum test with Bonferroni correction. (G). Expression of *Ace2* among type II pneumocytes binned by infection status in WT mice. All pairwise comparisons non-significant (p > 0.05) by Wilcoxon rank sum test. (H). Percent of *Ace2*^+^ cells by infection condition (uninfected, bystander cells in MHV68-infected mouse, MHV68 RNA+ cells) and mouse genotype (WT, IFNγR −/−). Black bars: *Ace2*^+^ positive cells; white bars: *Ace2*^-^ cells. (I). Schematic of RNA-Seq data from (Matos et al., 2019) of human lung explants (n = 5 donors) exposed to influenza A virus (IAV, H3N2) at 24 h post infection. (J). Expression of *SFTPC* (surfactant protein C, a marker of type II pneumocytes) versus *ACE2* among mock-infected lung explants. Statistical significance assessed by Pearson’s correlation, r = 0.93, p = 0.021. TPM: transcripts per million. (K). *SFTPC* expression among matched donors following mock or IAV infection for 24 h. Statistical significance assessed by ratio paired t test, p = 0.86. (L). *ACE2* expression among matched donors following mock or IAV infection for 24 h. Statistical significance assessed by ratio paired t test, p = 0.0054. (M). Western blot of fully-differentiated air-liquid interface cultures from bronchial cells derived from 4 human donors with asthma. Cells from each donor were treated with 10 ng/mL IFNγ for 24 h, and compared to a matched untreated condition. ACE2 protein: AF933 (R&D). Fold changes quantified for IFNγ treated versus untreated for each patient donor following normalization to GAPDH.

**Figure 6:**
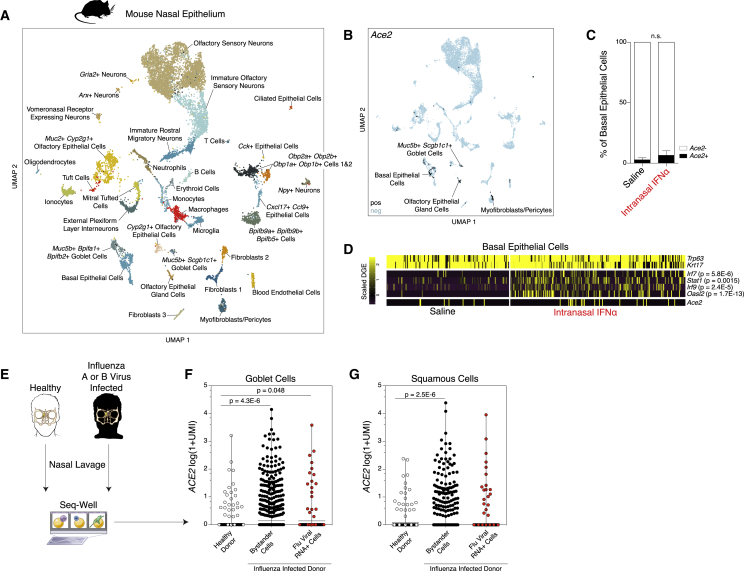
*In Vivo* Administration of Interferons in Mice Does Not Induce *Ace2*, and *ACE2* Is Induced in Goblet Secretory Cells during Human Influenza Infection (A) UMAP of 11,358 single cells from mouse nasal epithelium (n = 4). (B) UMAP projection as in (A), points colored by detection of *Ace2* (SARS-CoV-2 receptor homolog). Color coding is as follows: black, RNA positive; blue, RNA negative. (C) Percent of *Ace2*^+^ cells by treatment condition (n = 4 arrays per condition; n = 2 arrays per mouse). Black bars indicate *Ace2*^+^ cells; white bars indicate *Ace2*^*−*^ cells. p = 0.4 by Student’s t test. (D) Heatmap of cell-type-defining genes (*Trp63* and *Krt17)*, interferon-induced genes (*Irf7*, *Stat1*, *Irf9*, and *Oasl2*), and *Ace2* among basal epithelial cells, separated by cells derived from saline-treated mice (left) and IFN-α-treated mice (right). Statistical significance by likelihood-ratio test with Bonferroni correction is shown. A full list of differentially expressed genes can be found in Table S8. (E) Schematic for sampling cells derived from nasal washes of n = 18 human donors with and without current influenza A or B infection for Seq-Well v1 (35,840 single cells). See Cao et al., (2020). (F and G) *ACE2* expression among goblet cells (F) and squamous cells (G) by infection status. Shown are Healthy Donor cells from influenza-negative donors (white); Bystander Cells from influenza A (IAV)- or influenza B (IBV)-infected donors, no intracellular viral RNA detected (black); Flu Viral RNA^+^ Cells with detectable intracellular influenza A or B viral RNA (red). Statistical significance by Wilcoxon test with Bonferroni correction, n.s. for Bystander versus Flu Viral RNA^+^. See also Figure S5 and Tables S6 and S8.

Given minimal upregulation of *Ace2* among primary mouse basal cells *in vitro*, we were curious as to whether *Ace2* represented a murine ISG *in vivo*. We treated two mice intranasally with saline and two mice intranasally with 10,000 units of IFN-α (Guerrero-Plata et al., 2005). After 12 h, we isolated the nasal mucosa, consisting of both respiratory and olfactory epithelium, with underlying lamina propria, and performed scRNA-seq using Seq-Well S^3^ (Figure S5A). We collected from both tissue sites because of early reports of anosmia in COVID-19 (Lechien et al., 2020). We recovered 11,358 single cells, including epithelial, stromal, neuronal, and immune cell types, generating the largest single-cell atlas of mouse respiratory and olfactory mucosa to date (Figures 6A and S5B). We annotated all 36 clusters, focusing our attention on epithelial cell clusters, given that we noted enrichment for *Ace2* and *Tmprss2* within epithelial cell subsets, consistent with our human and NHP results (Table S8). Specifically, we found *Ace2* enriched within olfactory epithelial gland cells, *Muc5b*^+^*Scgb1c1*^+^ goblet cells, basal epithelial cells, and myofibroblasts/pericytes (Bonferroni-corrected p < 0.01) (Figures 6B and S5B) (Brann et al., 2020, Dear et al., 1991, Montoro et al., 2018, Tepe et al., 2018). Notably, *Furin* was enriched within olfactory epithelial gland cells (Table S8). Next, we asked whether a 12 h stimulation with IFN-α would upregulate *Ace2 in vivo.* Focusing on basal epithelial cells, which contain the highest abundance of *Ace2*^+^ cells, we found that despite robust upregulation of canonical murine ISGs, *Ace2* expression was only slightly elevated after IFN-α treatment (Figures 6C, 6D, S5C, and S5D).

This observation was supported by analysis of scRNA-seq data from 5,558 epithelial cells from the lungs of mice 3–6 days after intranasal infection with murine gamma herpesvirus-68 (MHV68) (Figure S5E). Here, we found significant enrichment of *Ace2*^+^ cells within type II pneumocytes, in line with our data from NHP and human lungs (Figures S5F). We did not observe changes in *Ace2* expression among viral-transcript-positive cells or “bystander” type II pneumocytes (those without detectable cell-associated viral RNA in MHV68-infected animals), nor did we see significant alterations in *Ace2*^+^ cell abundance among MHV68-infected mice lacking IFN-γR (Figure S5G and S5H). These observations were in agreement with our *in vitro* murine basal cell assay (Figure 5A and 5E).

Finally, we sought to validate our hypothesis that *ACE2* is upregulated in human epithelial cells during upper airway viral infections, which are known to induce a robust IFN response (Bailey et al., 2014, Everitt et al., 2012, Iwasaki and Pillai, 2014, Jewell et al., 2010, Russell et al., 2018, Steuerman et al., 2018). We re-analyzed a publicly available dataset of RNA-seq from human lung explants isolated after surgical resections that were infected with influenza A virus *ex vivo* for 24 h. Here, we found that *ACE2* expression was significantly correlated with that of *SFTPC*, supporting our hypothesis that *ACE2* is expressed within type II pneumocytes (Figures 1C, 2C, S5I, and S5J) (Matos et al., 2019). Furthermore, although the abundance of *SFTPC* was not significantly altered by influenza A virus infection, *ACE2* expression was significantly upregulated after viral exposure (p = 0.0054, ratio paired t test) (Figures S5K and S5L). This suggests that influenza A virus infection increases *ACE2* expression. Nevertheless, these population-level analyses are not able to definitively resolve specific cell subsets of relevance, nor whether they are directly infected cells or bystanders of infection.

In order to address these questions, we leveraged an ongoing scRNA-seq study of nasal washes from 18 individuals with confirmed influenza A virus or influenza B virus infection or healthy controls collected with Seq-Well v1, which yielded 35,840 cells resolved into 17 distinct cell types (Figure 6E; STAR Methods) (Cao et al., 2020). We investigated the cell types with greatest enrichment for *ACE2* and *TMPRSS2* in non-infected controls and individuals with influenza A and B. Strikingly, *ACE2* was most upregulated in samples from influenza-virus-infected individuals within bystander goblet or squamous cells not directly infected by virus (Figures 6F and 6G). *ACE2*^+^*TMPRSS2*^+^ goblet cells during influenza infection exhibited enrichment for canonical ISGs such as the *CXCL9/CXCL10/CXCL11* gene cluster; correspondence with *ACE2*^+^*TMPRSS2*^+^ goblet cells in healthy and allergic nasal scrapings; and a shared overlap in ISGs including *GBP2*, *ZNFX1*, *ADAR*, and *ACE2* (significantly differentially expressed gene lists) (Table S6). Together, our data suggest that *ACE2* is an ISG *in vitro* and *in vivo* in human primary upper airway epithelial basal cells, but that the murine homolog *Ace2* is not in airway epithelial basal cells or pulmonary epithelial cells *in vitro* or *in vivo*. Collectively, our findings suggest that careful considerations of animal and cellular models will be needed for assessing therapeutic interventions targeting the IFN system when studying *ACE2*/*Ace2*-associated biology.

Finally, because our *in vivo* and *in vitro* work indicate that IFN might promote human cellular targets for SARS-CoV-2 infection in the human upper airway by inducing *ACE2*, we attempted to extend our transcriptomic data on IFN-driven expression of *ACE2* to protein-level induction of ACE2. As testing of various commercially available polyclonal antibody preparations found broad evidence for non-specific or inconclusive staining in histological immunofluorescent based readouts (data not shown), we assessed whether IFN-γ-stimulated human bronchial air-liquid interface cultures induced ACE2 within 24 h. Our results show that cells from one patient robustly induced ACE2 (+2.02x), cells from another mildly induced ACE2 (+1.21x) and two patient’s cells showed minor changes (+/−1.12x) (Figure S5M). We provide a note of caution as these cells were derived from asthmatic patients, and the overall changes did not reach significance. Furthermore, we could not determine cell surface localization of ACE2 but do note that these results align with our transcriptomic data.

## Discussion

Here, we utilize scRNA-seq across various barrier tissues and model organisms to identify the potential initial cellular targets of SARS-CoV-2 infection. To review the data presented: (1) we found that expression of the cellular entry receptor for SARS-CoV-2, *ACE2*, is primarily restricted to type II pneumocytes in the lung, absorptive enterocytes within the gut, and goblet secretory cells of the nasal mucosa; (2) *ACE2* and *TMPRSS2* co-expression in respiratory tissues is consistently found only among a rare subset of epithelial cells; (3) we observed similarities in the cellular identities and frequencies of putative SARS-CoV-2 target cells across human and NHP cohorts; (4) we observe increased expression of *ACE2* during SHIV and TB infection of NHPs, and HIV/TB co-infection and influenza infection of humans compared with that in matched controls but caution that none of the datasets presented here were designed to answer this specific query. Specific targeting of these cell subsets has only been described for a handful of viruses, including the following: goblet cells by human adenovirus-5p and enterovirus 71, type II pneumocytes by H5N1 avian influenza, and absorptive enterocytes by rotavirus (Fleming et al., 2014, Good et al., 2019, Holly and Smith, 2018, Weinheimer et al., 2012).

**Figure S6:**
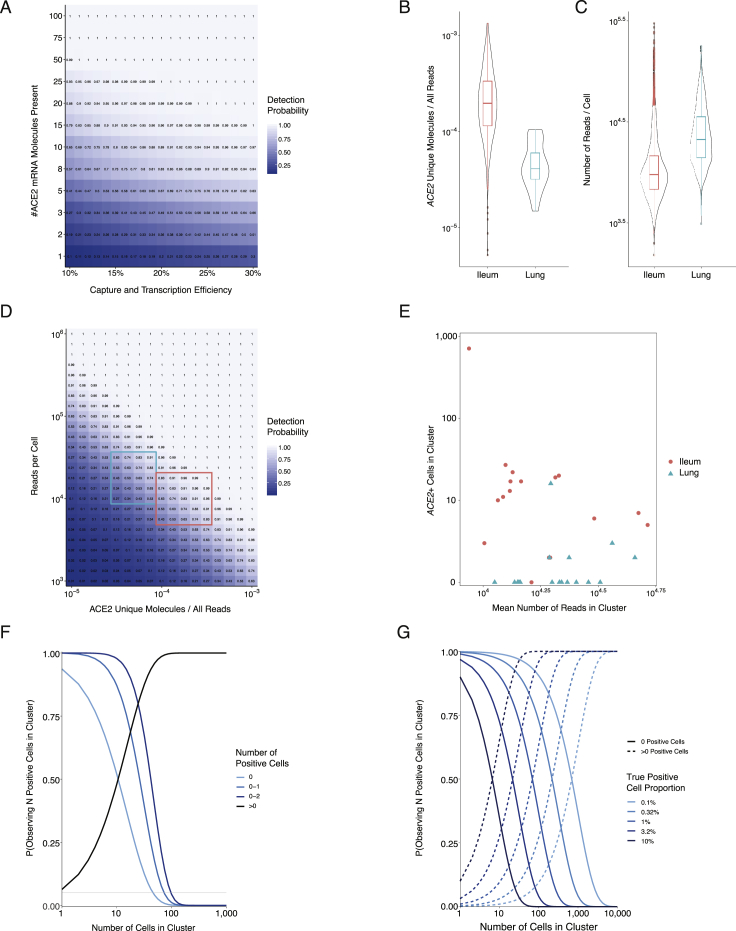
Power Calculations and Statistical Modeling of *ACE2* Capture and Dropout Related to STAR Methods (A). Probability of capturing and transcribing at least 1 *ACE2* cDNA molecule, as a function of the capture/reverse transcription efficiency for a single molecule and the number of *ACE2* molecules expressed in an individual cell. Note that Drop-Seq provides a capture/transcription efficiency of approximately 11-13%, setting a floor on this parameter, and the experimental platforms used in this study are either equivalent or superior (Macosko et al., 2015). (B). Distribution of *ACE2* fractional abundance within individual cells’ cDNA libraries (i.e., *ACE2* UMIs / total number of reads), across non-human primate lung and ileum cell populations (see Figures 1 and 3). Mean fractional abundance among *ACE2*^+^ lung cells = 5.0E-5; mean fractional abundance among *ACE2*^+^ ileum cells = 2.7E-4. (C). Distribution of the number of reads within non-human primate lung and ileum cell populations (see Figures 1 and 3). Mean ± SEM reads among all lung cells = 28,512 ± 344; *ACE2*^+^ lung cells = 28,553 ± 2,988; all ileum cells = 14,864 ± 288; *ACE2*^+^ ileum cells = 10,591 ± 441. (D). Probability of observing at least one transcript for a gene of interest (e.g., *ACE2*) within an individual cell, as a function of sequencing depth and the gene’s fractional abundance (i.e., *ACE2* reads / all reads) within the cell’s cDNA library. Fractional abundance provides the probability that a single read corresponds to the gene of interest, and presented heatmap indicates the probability that at least one read in the total number of reads allocated to the cell (i.e., from 10^3^ to 10^6^) originates from the gene of interest. Mean read depths and *ACE2* fractional abundances for each tissue produce a 93.7% probability of detecting at least 1 *ACE2* read in ileum cells, and a 76.0% chance for lung cells. Outlined rectangles highlight the regimes where cells from lung (turquoise) and ileum (pink) samples typically lie. (E). Number of *ACE2*^+^ cells within each cluster, as a function of average read depth for all cells in that cluster. Number of cells detected as *ACE2*^+^ is not correlated with read depth, even across relatively wide ranges of average read depths (Pearson’s r = −0.31, n.s.). (F). Probability of observing a particular number of cells positive for a gene of interest within a cluster, as a function of number of cells in the cluster. Probabilities were calculated under a negative binomial distribution with parameter p = 0.063 (the proportion of *ACE2*^+^ cells among type II pneumocytes presented in Figure 1; STAR Methods). The horizontal gray line indicates the arbitrary cut-off value of p = 0.05. (G). Given a population of cells with a known proportion that are positive for a gene of interest, probability of observing no positive cells (i.e., false negative identification of the cluster; solid lines) and probability of observing at least one positive cell as a function of cluster size.

Additionally, we provide an overall note of caution when interpreting scRNA-seq data for low abundance transcripts like *ACE2* and *TMPRSS2* because detection inefficiencies might result in an underestimation of the actual frequencies of *ACE2*^+^ or *ACE2*^+^*TMPRSS2*^+^ cells in a tissue. Moreover, the protein amounts of each might differ from their mRNA abundances (Genshaft et al., 2016, Jovanovic et al., 2015, Rabani et al., 2011, Shalek et al., 2013). We also present datasets separately, given that each study differed in its methods of tissue processing and collection, which can influence the frequency of recovered cell subsets (STAR Methods). We provide Table S9 as a summary of *ACE2*^+^ and *ACE2*^+^*TMPRSS2*^+^ cells across various datasets. Moreover, we present Figure S6, which describes statistical modeling and power calculations underlying detection and dropout of *ACE2*, to help guide interpretation of these data. This includes an examination of the probability to detect a lowly expressed transcript like *ACE2* within a cell, as well as upper bound estimates on the percentage of positive cells within a cluster, considering the effects of transcript counts, sequencing depth, and cell numbers in these calculations (STAR Methods).

Whether ACE2 and TMPRSS2 are needed on the same cell or soluble proteases can activate SARS-CoV-2 S protein to invade ACE2 single-positive cells is an area of active inquiry (Coutard et al., 2020, Letko et al., 2020). Importantly, rapidly evolving literature has identified that SARS-CoV-2-S might have a furin cleavage site, leading to a broader set of host proteases that could mediate S protein activation (Bugge et al., 2009, Coutard et al., 2020, Walls et al., 2020). However, because an active S protein has a finite lifetime to find a target cell membrane, the timing and cellular location of S protein activation is key to consider. Activation events proximal to the plasma membrane have been shown to be most effective for SARS-CoV entry (Shulla et al., 2011).

Our study finds that type I IFNs, and to a lesser extent type II IFNs, upregulate *ACE2.* This is based on several lines of evidence: (1) we identified a human goblet secretory cell subset in upper airway nasal epithelium enriched for *ACE2* expression to have the highest IFN-α-induced gene signature; (2) we found that IFN-α, and to a lesser extent IFN-β or IFN-γ, induced *ACE2* expression in a published dataset of air-liquid interface cultures derived from human nasal epithelial cells (Giovannini-Chami et al., 2012, Ordovas-Montanes et al., 2018); (3) we extended our search through the Interferome database (Rusinova et al., 2013) and found that, in epithelial barrier tissues, type I IFNs upregulate *ACE2* in multiple studies, especially in primary bronchial cells and keratinocytes (Rusinova et al., 2013); (4) we found two STAT1 binding sites in the promoter of *ACE2*; (5) in our unpublished atlas of SHIV-infected macaques, known to have elevated amounts of chronic IFN signaling, we found *ACE2* upregulation in absorptive enterocytes; (6) we directly provided evidence for IFN-α, and to some extent IFN-γ, inducing *ACE2* expression in primary human upper airway basal cells; and (7) influenza infection in humans, a known inducer of the IFN pathway, leads to increased *ACE2* expression in goblet secretory cells of the nasal epithelium (Cao et al., 2020).

Altogether, our own and publicly available data highlight that *ACE2* might have been missed as a canonical ISG because of its notable absence in peripheral blood mononuclear cell datasets and in lung-derived transformed cell lines such as the A549 cell line (Butler et al., 2018, Letko et al., 2020, Rusinova et al., 2013). Importantly, other groups have independently analyzed publicly available datasets, some referenced in our work, and observed ACE2’s behavior as an ISG (Wang and Cheng, 2020). Furthermore, we found weak IFN- or virally driven induction of *Ace2* in murine cells and tissues. This highlights the importance of studying primary human epithelial cells and the careful consideration of appropriately selected gene lists and *in vitro* models of *in vivo* cellular systems for understanding human biology (Jonsdottir and Dijkman, 2016, Mead and Karp, 2019, Regev et al., 2017).

As SARS-CoV-S leads to ACE2-receptor-mediated internalization, the host IFN response could thus promote the ability for SARS-CoV and SARS-CoV-2 to maintain cellular targets in neighboring human upper airway epithelial cells. Altogether along with a study of HCoV-OC43, which co-opts IFN-inducible transmembrane 2 (IFITM2) and IFITM3 to promote viral entry, this adds to the growing evidence that coronaviruses, as well as other viruses, have evolved to leverage features of the human IFN pathway (Fung and Liu, 2019, Mar et al., 2018, Zhao et al., 2014). Whether type I IFNs are net protective or detrimental to the host might depend on the stage of infection; cell subsets in question; the SARS viral clade (Channappanavar et al., 2016, Channappanavar et al., 2019, Channappanavar and Perlman, 2017, Davidson et al., 2015); and other factors such as co-infection, age, gender, and co-morbidities, among others. Understanding the specific host restriction factors targeting SARS-CoV-2 and identifying specific drivers of these genes in the absence of *ACE2* upregulation might provide strategies to dissociate the dual roles of IFN in certain coronavirus infections. Whether IFNs upregulate ACE2 in putative target cell subsets *in vivo* will be of significant interest to define in future work once current COVID-19-related restrictions on basic scientific inquiry are lifted (Qian et al., 2013).

ACE2 is a central component of the renin-angiotensin system, which has emerged as a key regulator of sterile- or microbially induced lung pathology (Imai et al., 2005). In brief, ACE cleaves angiotensin I to generate angiotensin II (Skeggs et al., 1980). Angiotensin II then acts to drive acute lung injury through various mechanisms, including increased vascular permeability (Imai et al., 2005). Amounts of angiotensin II in humans and mice are elevated during influenza infection, and ACE2 exerts tissue-protective functions by reducing amounts of angiotensin II (Zou et al., 2014). Binding of SARS-CoV-S to mouse ACE2 *in vivo* reduced ACE2 expression leading to acute acid-aspiration-induced lung failure (Kuba et al., 2005). Depending on the questions asked in future work, there are mouse models available on the basis of transgenic expression of human ACE2 (required for overt infectious pathology of SARS-CoV in mice), there are established NHP models available of SARS-CoV infection in *M. fascicularis* and *C. aethiops*, and early reports suggest symptomatic infection in *M. mulatta* and *M. fascicularis* models for SARS-CoV-2 (Bao et al., 2020, McCray et al., 2007, Munster et al., 2020, Rockx et al., 2020, Smits et al., 2011). For example, examining the efficacy of recombinant human ACE2 to act as a decoy receptor or the effect of “ACE inhibitors” in patients with, or at risk for, COVID-19 will require careful experimentation in appropriate models together with well-controlled clinical trials (Hofmann et al., 2004, Monteil et al., 2020, Vaduganathan et al., 2020).

IFN responses that induce ISGs are essential for host antiviral defense in mice, NHPs, and humans (Bailey et al., 2014, Dupuis et al., 2003, Everitt et al., 2012). Canonical ISGs function by directly restricting viruses and reducing burden (Schneider et al., 2014). More recently, disease tolerance to equivalent pathogen burden by factors that increase the ability of the host to tolerate tissue damage has been identified as part of a combined host defense strategy (Iwasaki et al., 2017, Iwasaki and Pillai, 2014, Medzhitov et al., 2012, Schneider and Ayres, 2008). Disease tolerance factors in the lung include IL-22 and amphiregulin (Iwasaki et al., 2017). During acute infection in the respiratory system, ACE2 is critical for early tissue tolerance responses to respiratory infection, including H5N1 influenza (Huang et al., 2014, Zou et al., 2014). However, our discovery that *ACE2* is an ISG in human epithelial cells, along with SARS-CoV-2 utilizing host ACE2 to gain entry to cells, suggests that SARS-CoV and SARS-CoV-2 might exploit the ACE2-mediated tissue-protective response to provide further cellular targets for entry. This potential strategy employed by SARS-CoV-2 could present a unique challenge for the human host and is distinct from HCoV-OC43, which targets the two restriction factors IFITM2 and IFITM3 (Zhao et al., 2014). Our study provides motivation to understand the specific role and balance of type I and type II IFNs, as well as type III IFNs, in tissue protection during, and host restriction of, SARS-CoV-2 infection. Key experiments to understand *ACE2* as an ISG in tissue protection or genuine tolerance will require the appropriate mouse, NHP, or other model in BSL3 or BSL4 facilities to execute SARS-CoV-2 viral infections and measure host tissue health along with viral loads. Further work will also be needed to understand how co-infections, as well as other host factors, might affect both the susceptibility to, and dynamics of, host SARS-CoV-2 infection. Moreover, carefully controlled clinical trials will be essential to determine the overall effects of different IFNs (Prokunina-Olsson et al., 2020).

Altogether, we anticipate that comprehensive characterization of the putative cellular targets of SARS-CoV-2 will be critical to understand basic mechanisms of viral tropism and disease pathophysiology, inform differential susceptibility among vulnerable populations, and potentially suggest unanticipated targets for drug inhibitors of viral infection. The cellular targets we nominate will need to be confirmed by specific reagents for SARS-CoV-2, as done for SARS-CoV (Ding et al., 2004). Furthermore, the transcriptional response to the virus will need to be rigorously characterized in appropriate *in vitro* and *in vivo* model systems (Blanco-Melo et al., 2020). We provide gene lists associated with target cells in specific tissues and diseases to aid the community in understanding this emergent disease. A concurrent HCA Lung Biological Network study assessing *ACE2* and *TMPRSS2* across more tissues also identified enrichment in nasal goblet and ciliated cells (Sungnak et al., 2020). Other studies are considering additional tissues; co-variates such as age, sex, and co-infection state; and represent a large coordinated international effort to the ongoing crisis (Pinto et al., 2020). One study in particular identified upregulation of *ACE2* by respiratory viruses and *TMPRSS2* by IL-13 in a pediatric cohort, suggesting further links to how underlying allergic conditions or co-infections might modulate these two SARS-CoV-2-related host factors (Sajuthi et al., 2020).

During the preparation of this manuscript, several papers have been posted to bioRxiv assessing patterns of *ACE2*^+^ and *TMPRSS2*^+^ cells in barrier tissues (Brann et al., 2020, Lukassen et al., 2020, Qi et al., 2020, Wu et al., 2020, Zhang et al., 2020). At a high level, these studies are largely in agreement with our report. Furthermore, another study appeared on medRxiv profiling bronchoalveolar lavage fluid from 3 severe and 3 mild COVID-19 patients, though they were unable to profile sufficient numbers of epithelial cells (Liao et al., 2020).

Our study highlights the power of scRNA-seq datasets, both existing and novel, to derive hypotheses relevant to human disease that might differ from paradigms established by using cell lines. Further work will be critical to determine how SARS-CoV-2 influences temporal dynamics of host responses at single-cell resolution and which host factors might affect this (Kazer et al., 2020). Given the unappreciated complexities of host-pathogen interactions between humans and SARS-CoV-2, the best measures to combat this pandemic continue to be surveillance and avoidance—especially given that a deep understanding of the full spectrum of resistance and tolerance mechanisms will require the concerted efforts of scientists around the globe (Amanat et al., 2020, Chu et al., 2020, Hadfield et al., 2018). Here, we seek to share our initial findings and data so that other groups might build on this discovery of *ACE2* as an ISG and further consider the careful balance between tissue tolerance and viral infection needed at the human airway epithelium.

## STAR★Methods

### Key Resources Table

**Table:** 

REAGENT or RESOURCE	SOURCE	IDENTIFIER
**Biological Samples**		

*M. mulatta* lung, bone marrow, brain, colon, ileum, jejunum, liver, lung, peripheral blood, spleen, thymus, tonsil, and lymph nodes from various sites	Washington National Primate Research Center	N/A
Human lung tissue from surgical excess	University of KwaZulu-Natal	IRB Code: BE024/09
Human non-inflamed ileal pinch biopsies	Multi-center clinical study, approved by the Institutional Review Board at Boston Children’s Hospital	IRB Code: IRB-P00030890
Human nasal lavage	University of Massachusetts Medical School	N/A
Human nasal scraping, polyp scrapings, ethmoid sinus surgical tissue samples	Partners HealthCare Institute	N/A
*M. fascicularis* lung and granulomatous tissue	University of Pittsburgh School of Medicine	N/A

**Antibodies**		

anti-ACE2 human antibody, goat polyclonal	R&D	Cat#AF933

**Bacterial and Virus Strains**		

MHV-68	Adler et al., 2000	N/A
*Mycobacterium Tuberculosis*, Modified Erdman Strain	Martin et al., 2017	N/A

**Chemicals, Peptides, and Recombinant Proteins**		

2-Mercaptoethanol	Sigma	Cat#M3148-25ML
RLT Buffer	QIAGEN	Cat#79216
dNTP	New England BioLabs	Cat#N0447L
RNase Inhibitor	Fisher Scientific	Cat#AM2696
Maxima RNaseH-minus RT Enzyme	Fisher Scientific	Cat#EP0753
MgCl_2_	Sigma	Cat#63069-100ML
Betaine	Sigma	Cat#B0300-5VL
AMPure RNAClean XP RNA-SPRI beads	Beckman Coulter	Cat#A63987
AMPure XP SPRI beads	Beckman Coulter	Cat#A63881
Guanidinium thiocyanate	Sigma	Cat#AM9422
Sarkosyl	Sigma	Cat#L7414
Exonuclease I	New England BioLabs	Cat#M0293S
Klenow Fragment	New England BioLabs	Cat#M0212L
DNase I	Roche	Cat#10104159001
Collagenase IV	Life Technologies	Cat#17104019
Collagenase D	Roche	Cat#11088858001
Liberase TM	Roche	Cat#5401119001
TrypLE	Thermo Fisher	Cat#12604013
ACK Buffer	Thermo Fisher	Cat#A1049201
IFN-α	Biolegend	Cat#752802
Dispase II	Thermo Fisher	Cat#17105041
Elastase	Worthington Biochem	Cat#LS002292
Pneumacult-Ex serum-free media	StemCell Technologies, Inc.	Cat#05040
IL-4, human	Biolegend	Cat#574002
IL17A, human	Biolegend	Cat#570502
IFNγ, human	Biolegend	Cat#570202
IFNγ, mouse	Peprotech	Cat#315-05
IFNα, human	Biolegend	Cat#592702
IFNα, mouse	Biolegend	Cat#752802
IFNβ, mouse	R&D Systems	Cat#8234-MB-010

**Critical Commercial Assays**		

Nextera XT DNA Library Preparation Kit	Illumina	Cat#FC-131-1096
High Sensitivity D5000 ScreenTape	Agilent	Cat#5067-5592
Qubit dsDNA High-Sensitivity kit	ThermoFisher	Cat#Q32854
NextSeq 500/550 High Output v2 (75 cycles)	Illumina	Cat#FC-404-2005
NovaSeq 6000 S2 (100 cycles)	Illumina	Cat#20012862
Kapa HiFi HotStart ReadyMix	Kapa Biosystems	Cat#KK2602
MACOSKO-2011-10 mRNA Capture Beads	ChemGenes	Cat#NC0927472
Tumor Dissociation Kit, Human	Miltenyi Biotec	Cat#130-095-929
Chromium Single Cell 3′ v2	10X Genomics	Cat#120237

**Deposited Data**		

scRNA-seq Processed Data	This paper	https://singlecell.broadinstitute.org/single_cell?scpbr=the-alexandria-project
scRNA-seq Processed Data	This paper	https://drive.google.com/drive/folders/1bxCIqNeZ7wLuVOT16gphwj98_cc9KhrV?usp=sharing
scRNA-seq Processed Data	This paper	https://chanzuckerberg.github.io/cellxgene/posts/cellxgene_cziscience_com
scRNA-seq Processed Data	This paper	https://singlecell.broadinstitute.org/single_cell/covid19
scRNA-seq Processed Data (all species) and FASTQ files (for NHP and murine datasets)	This paper	GEO: GSE148829
scRNA-seq data from human nasal mucosa	Ordovas-Montanes et al., 2018	https://singlecell.broadinstitute.org/single_cell/study/SCP253/allergic-inflammatory-memory-in-human-respiratory-epithelial-progenitor-cells
Human reference genome NCBI build 38 (GRCh38)	Genome Reference Consortium	http://www.ncbi.nlm.nih.gov/projects/genome/assembly/grc/human/
Human reference genome NCBI build 19	Genome Reference Consortium	http://www.ncbi.nlm.nih.gov/projects/genome/assembly/grc/human/
Mouse reference genome NCBI build 10	Genome Reference Consortium	http://www.ncbi.nlm.nih.gov/projects/genome/assembly/grc/mouse/
*Macaca mulatta* reference genome assembly 8.0.1, annotation 102	NCBI Eukaryotic Genome Annotation Pipeline	https://www.ncbi.nlm.nih.gov/genome/annotation_euk/Macaca_mulatta/102/
*Macaca fascicularis* reference genome assembly 5, annotation 101	NCBI Eukaryotic Genome Annotation Pipeline	https://www.ncbi.nlm.nih.gov/genome/annotation_euk/Macaca_fascicularis/101/
Interferome Database	Rusinova et al., 2013	http://www.interferome.org/
RNA-seq from human lung explants ± *ex vivo* IAV infection	Matos et al., 2019	GEO: GSE135069
RNA-seq from human nasal epithelial cells	Giovannini-Chami et al., 2012	GEO: GSE19190, GSE22147

**Experimental Models: Cell Lines**		

Human: Passage 4 BEAS-2B	ATCC	CRL-9609

**Experimental Models: Organisms/Strains**		

Mouse: C57BL/6J	The Jackson Laboratory	Cat#000664
Mouse: C57BL/6, IFNγR−/− B6.129S7-*Ifngr1*^*tm1Agt*^/J	The Jackson Laboratory	Cat#003288

**Oligonucleotides**		

SMART-seq2 2 3′ Oligo-dT Primer: /5Biosg/AAG CAG TGG TAT CAA CGC AGA GTA CTT TTT TTT TTT TTT TTT TTT TTT TTT TTT TVN	Integrated DNA Technologies	N/A
SMART-seq2 5′ TSO: AAG CAG TGG TAT CAA CGC AGA GTA CAT rGrGrG	Integrated DNA Technologies	N/A
SMART-seq2 and Seq-Well ISPCR: AAG CAG TGG TAT CAA CGC AGA GT	Integrated DNA Technologies	N/A
Custom Read 1 Primer: GCC TGT CCG CGG AAG CAG TGG TAT CAA CGC AGA GTA C	Integrated DNA Technologies	N/A
Seq-Well 5′ TSO: AAG CAG TGG TAT CAA CGC AGA GTG AAT rGrGrG	Integrated DNA Technologies	N/A
Seq-Well Custom P5-SMART PCR hybrid oligo: AAT GAT ACG GCG ACC ACC GAG ATC TAC ACG CCT GTC CGC GGA AGC AGT GGT ATC AAC GCA GAG TAC	Integrated DNA Technologies	N/A
Seq-Well dN-SMRT oligo: AAG CAG TGG TAT CAA CGC AGA GTG ANN NGG NNN B	Integrated DNA Technologies	N/A

**Software and Algorithms**		

R	R Core Team	https://www.r-project.org
R package – Seurat v2.3.4 and v3.1.0	Github	https://github.com/satijalab/seurat
Scanpy	Wolf et al., 2018	https://github.com/theislab/scanpy
R package – SCDE	Bioconductor	http://bioconductor.org/packages/scde/
Prism 6	GraphPad Software	https://www.graphpad.com/scientific-software/prism/
STAR	Github	https://github.com/alexdobin/STAR
Uniform Manifold Approximation and Projection	Github	https://github.com/lmcinnes/umap
Rtsne	CRAN	https://cran.r-project.org/web/packages/Rtsne/

### Resource Availability

#### Lead Contact

Further information and requests for resources and reagents should be directed to and will be fulfilled by Dr. Jose Ordovas-Montanes (jose.ordovas-montanes@childrens.harvard.edu).

#### Materials Availability

This study did not generate new unique reagents.

#### Data and Code Availability

In Table S9**,** we provide a guide to all datasets analyzed in this paper as well as links to each individual dataset for download with the main landing page here: https://singlecell.broadinstitute.org/single_cell?scpbr=the-alexandria-project. To download the data from the portal, follow the link to the visualization page, sign in a free account in the portal using a Google apps enabled email address, and select the ‘Download’ tab in the study. Downloadable datasets include both raw and normalized cell x gene matrices, as well as relevant metadata. These datasets are additionally available here to facilitate downloading: https://drive.google.com/drive/folders/1bxCIqNeZ7wLuVOT16gphwj98_cc9KhrV?usp=sharing. We have also posted these cell x gene matrices to Chan Zuckerberg Initiative cellxgene (https://chanzuckerberg.github.io/cellxgene/posts/cellxgene_cziscience_com) and the Broad Institute Single Cell COVID-19 portal (https://singlecell.broadinstitute.org/single_cell/covid19) as leading community efforts. FASTQ files and cell x gene matrices for NHP and murine datasets, and cell x gene matrices for human datasets, are available at GEO: GSE148829.

In this same table, we further highlight four access types. 1. published datasets where everything is available (1 study); 2. unpublished datasets where everything is available (2 studies, 19,670 new cells for download), 3. unpublished datasets where *ACE2*+ cell subsets, and the necessary subsets to contextualize those cells (i.e., epithelial cells for type II pneumocytes) are fully available (5 studies, 17,986 new cells for download); and, 4. those unpublished datasets where expression is shared for *ACE2/TMPRSS2* (2 studies, 9,112 new cells). For those unpublished datasets where only specific subsets of cells or genes are available, full expression matrices are available upon request for COVID-19 related questions.

All data included in the present study can be visualized using the following web viewer:

https://singlecell.broadinstitute.org/single_cell?scpbr=the-alexandria-project.

As we gain further insight and feedback from our own groups, collaborators, and investigators, we will continue to provide updates on our resource websites, including the utility of *in vitro* systems, such as organoids (Mead et al., 2018), for the study of SARS-CoV-2: http://shaleklab.com/resource/covid-19-resources/ and www.ordovasmontaneslab.com/covid-19-resources/. We also note that there are several ongoing efforts unified together through the HCA Lung Biological Network group that we will reference and to which we will link as they become available.

No custom code was used to analyze these data and all methods and packages used are cited in the Method Details section.

### Experimental Model and Subject Details

#### Human Intestinal Biopsies

For human intestinal biopsies from the terminal ileum, the subjects were enrolled on a multi-center clinical study, which was approved by the Institutional Review Board at Boston Children’s Hospital (protocol number: IRB-P00030890). Full information related to subject age/developmental stage and sex found in metadata associated with provided raw datasets.

#### Human Lungs, Surgical Excess

Samples were obtained through indicated lung lobe resection or diagnostic procedures in collaboration with clinicians at the Department of Cardiothoracic Surgery at Inkosi Albert Luthuli Central Hospital in Durban, South Africa. Informed consent was obtained from each participant. The study protocol was approved by the University of KwaZulu-Natal Institutional Review Board (approval BE024/09). Full information related to subject age/developmental stage and sex found in metadata associated with provided raw datasets.

#### Human Nasal Polyps and Scrapings

For inferior turbinate nasal scrapings, polyp scrapings, and ethmoid sinus surgical tissue samples, the Partners HealthCare Institutional Review Board (Boston, Massachusetts), approved the study and all subjects provided written informed consent (Ordovas-Montanes et al., 2018). Full information related to subject age/developmental stage and sex found in metadata associated with provided raw datasets.

#### Human Nasal Washes, Healthy and Influenza Infected

The Institutional Review Board of the University of Massachusetts Medical School (Worcester, Massachusetts) approved the study and all subjects provided written informed consent.

#### Cell Culture of Primary Basal Cells and Cell Lines

Human basal cells from non-polyp surgical resections from ethmoid sinus, BEAS-2B cells (ATCC), or mouse tracheal basal cells were placed into culture at a number of 10,000 cells seeded at passage 3 and cultured at confluence in 96 well flat-bottom collagen-coated tissue culture plates (Corning 3596) for 48 h in Pneumacult-Ex serum-free media (StemCell Technologies, Inc.). All cells were incubated at 37°C and 5% CO2.

#### Non-Human Primates (*M. mulatta*)

Healthy and SHIV-infected non-human primate (*M. mulatta)* work was conducted at the Washington National Primate Research Center (WaNPRC), an AAALAC accredited program, in accordance with the regulations detailed in the U.S. Department of Agriculture Animal Welfare Act and in the Guide for the Care and Use of Laboratory Animals of the National Institutes of Health. It was approved by University of Washington Institutional Animal Care and Use Committee. Expanded cohort characteristics described previously (Colonna et al., 2018). Full information related to subject age/developmental stage and sex found in metadata associated with provided raw datasets.

#### Non-Human Primates (*M. fascicularis*)

Tissues from *Mycobacterium tuberculosis*-infected non-human primates (*M. fascicularis)* were conducted at the University of Pittsburgh School of Medicine, an AAALAC accredited program, in accordance with the regulations detailed in the U.S. Department of Agriculture Animal Welfare Act and in the Guide for the Care and Use of Laboratory Animals of the National Institutes of Health. Full information related to subject age/developmental stage and sex found in metadata associated with provided raw datasets.

#### Mouse Nasal and Olfactory Epithelium and Tracheal Cells

C57BL/6J mice purchased from Jackson laboratory (Bar Harbor, ME, USA) were maintained within Ragon Institute’s HPPF barrier facility and all experiments were conducted with institutional IACUC approval. In this study, mice were 8-10 weeks of age, representing male and female animals.

#### Mouse Lungs, MHV68 Infection

C57BL/6 mice were purchased from Charles River Laboratories (Sulzfeld, Germany). IFNγR−/− mice on C57BL/6 background (C57BL/6, IFNγR^−/−^ B6.129S7-*Ifngr1*^tm1Agt^/J) were originally obtained from the Jackson Laboratory (Bar Harbor, ME, USA) and subsequently bred and propagated under SPF conditions at the Helmholtz Zentrum München. Animals with different genotypes were kept in the same animal room for the time of the experiment including an adaptation period prior to the start of the experiment. All animal experiments were in compliance with the German Animal Welfare Act (German Federal Law §8 Abs. 1 TierSchG), and the protocols were approved by the local Animal Care and Use Committee.

### Method Details

#### Methods of Sample Collection and Tissue Preparation for Single-Cell RNA-Seq

##### NHP Ileum, Jejunum, Colon, Liver, Tonsil, Thymus, and Lung Tissue

Animals were perfused with 0.5 L of PBS/kg immediately following euthanasia, tissues were isolated and placed in RPMI + 10% FBS and kept on ice until dissociation. Tissue sections were digested by mincing and incubating with collagenase IV (Life Technologies) and DNase I (Roche) at 37°C for 1 h with agitation. Digested tissue was passed through a 100 μm metal strainer, cells were pelleted by centrifugation at 300 g, rinsed with RPMI + 10% FBS, counted, and prepared as a single cell suspension for scRNA-seq using Seq-Well v1 (see below).

##### NHP Lymphoid Organs, Bone Marrow, PBMCs

All lymph nodes, spleen, and bone marrow were ground through a metal strainer, transferred to a conical in RPMI + 10% FBS, and pelleted by centrifugation at 400 g x 10 min. LN-derived cells were resuspended in RPMI + 10% FBS, counted and prepared as a single cell suspension. Spleen, bone marrow, and PBMCs were subjected to ACK lysis for 10 min at room temperature, quenched with RPMI + 10% FBS. PBMCs and bone marrow derived cells were purified over a ficoll gradient (GE Healthcare) by centrifuging at 400 g for 20 min at room temperature with no brake. Cells were then resuspended in RPMI + 10% FBS, counted, and diluted for scRNA-seq using Seq-Well v1 (see below).

##### NHP Tuberculosis Infected Lung and Granuloma

Ten *Mycobacterium tuberculosis* infected (Martin et al., 2017) adult non-human primates (*M. fascicularis*) were included in this study. A piece of lung tissue (without any grossly visible pathology) and 4 individual TB lung granulomas per animal were excised at necropsy and enzymatically dissociated using the GentleMacs system (Tumor dissociation kit, human; Miltenyi Biotec). Single cell suspensions were resuspended in RPMI + 10% FBS, counted and diluted for scRNA-seq using Seq-Well S^3^ (see below).

##### Human Lung Tissue

Surgical samples from diseased lung tissue (n = 3 TB^+^HIV^+^; n = 3 TB^+^; n = 2 non-infected patients) were processed as described in (Ardain et al., 2019). Briefly, each sample was collected into cold RP-10 (RPMI (Sigma-Aldrich) + 10% FBS), minced, and incubated for 25-30 min at 37°C with digestion buffer containing collagenase D (Sigma-Aldrich), DNase I (Sigma-Aldrich) in RPMI 1640 (Sigma-Aldrich) with 10% FBS (Hyclone). Following incubation, samples were homogenized using a GentleMACS, filtered using a 70 μm metal strainer, and pelleted by centrifugation at 400 g for 5 min. After obtaining the pellet, cells were resuspended in RP-10, passed through another 70μm strainer (Corning), stained with trypan blue, and then counted and diluted for scRNA-seq using Seq-Well S^3^ (see below).

##### Human Ileum

Single-cell suspensions were collected from biopsies as described (Smillie et al., 2019). Briefly, biopsies were rinsed in cold PBS, the epithelial layer was separated from the underlying lamina propria by end over end rotation for 15 min. The lamina propria and epithelial fractions were digested separately, using Liberase TM (Roche) and DNase I (Roche) for the lamina propria, and TrypLE (ThermoFisher) for the epithelial fraction. Following digestion, cells were pelleted by centrifugation, subjected to ACK lysis for 3 min, and filtered through a 40 μm strainer. Following centrifugation, the cells were counted and prepared as a single cell suspension for scRNA-seq using 10X 3′ v2 (10X Genomics).

##### Nasal Mucosa and Nasal Scrapings

Surgical samples from ethmoid sinus and nasal scraping of the inferior turbinate were processed as described (Ordovas-Montanes et al., 2018). Briefly, each sample was collected into cold RPMI (Corning), minced and incubated for 30 min (15 min for nasal scrapings) at 37°C with digestion buffer containing collagenase IV (Worthington), DNase I (Roche) in RPMI with 10% FBS. Samples were triturated and digestion quenched with EDTA. Cells were filtered using a 70 μm metal strainer and pelleted by centrifugation at 500 g, rinsed with PBS, and subjected to red blood cell (RBC) lysis using ACK buffer (ThermoFisher) for 3 min on ice, and finally pelleted prepared as a single cell suspension for scRNA-seq using Seq-Well v1 or S^3^ (see below).

##### Interferon Treatment of Mouse Nasal Mucosa

Mice received either 200ng of IFNα (Biolegend 752802) or saline intranasally (each group n = 2 mice), and were sacrificed 12 h later. Respiratory and olfactory mucosa were isolated as in (Davidson et al., 2004, Dunston et al., 2013). Briefly, using surgical tools under a dissecting microscope, the skull bones surrounding the nasal tissue of skinned mouse heads were removed. The respiratory and olfactory mucosa were collected in RPMI media with 10% FBS. Cells were digested in media containing Liberase TM (Roche) and DNase I (Roche) for 30 min at 37°C with agitation. Cells were filtered using a 70 μm strainer, washed with EDTA-containing media to quench enzymatic digestion, and pelleted by centrifugation. RBCs were lysed using ACK buffer (ThermoFisher) for 2 min, cells were again pelleted, counted, and prepared as a diluted single cell suspension for scRNA-seq using Seq-Well S^3^.

##### MHV68 Infected Mouse Lung

Mice were housed in individually ventilated cages during the MHV68 infection period. MHV68 stocks were grown and quantified by plaque assay as previously described (Adler et al., 2000). Mice were infected intranasally (i.n.) with 5 × 10^∗^4 plaque forming units of MHV68 diluted in PBS in a total volume of 30 μl. Prior to i.n. infection, mice were anesthetized with medetomidine–midazolam–fentanyl. At the predetermined time points, mice were sacrificed by cervical dislocation and lung tissue was processed for subsequent experiments. All lobes were removed, minced and transferred for mild enzymatic digestion for 20-30 min at 37°C in an enzymatic mix containing Dispase (50 caseinolytic U/mL), Collagenase (2 mg/mL), Elastase (1 mg/mL), and DNase I (30 μg/mL). Single cells were harvested by straining the digested tissue suspension through a 70μm strainer. After centrifugation at 300 x g for 5 min, single cells were counted, and prepared as a single cell suspension. For Drop-seq, cells were aliquoted in PBS supplemented with 0.04% of bovine serum albumin at a final concentration of 100 cells/μl.

##### Nasal Washes during Influenza Infection

Nasal washes were obtained from adult healthy controls and from adults with diagnosis of acute influenza A or B by rapid antigen test (Flu A or B antigen, direct fluorescence antigen test) and/or by respiratory virus panel (PCR testing for influenza A, influenza A H1, influenza A H3, influenza B, adenovirus, metapneumovirus, respiratory syncytial virus A, respiratory syncytial virus B, rhino/enterovirus, parainfluenza 1, parainfluenza 2, parainfluenza 3), who show symptoms up to seven days (Cao et al., 2020). Samples were obtained by irrigation of each naris with up to 10 mL of saline, and collected in a single container. The sample was then transported to the research laboratory for processing. Upon receipt, the sample was immediately stored on ice and 10 mL cell growth media (DMEM or RPMI1640 with 10% fetal bovine serum) was added. The material was strained using a 40 μm nylon cell strainer (Corning) into a 50 mL centrifuge tube. Cells were pelleted at 1300 rpm for 10 min at 4°C. All but 1 mL of supernatant was discarded, the pellet resuspended in the remaining 1 mL of supernatant, and material was transferred to an Eppendorf tube and pelleted at 2000 rpm for 5 min. If the pellet contained visible blood, 200 μL of RBC lysis solution (ACK buffer, Thermo Fisher) was added to resuspend the pellet and incubated at room temperature for 2 min, after which 1 mL of cell media was added, and the cells were pelleted at 2000 rpm for 5 min. The final pellet was resuspended in up to 1 mL of media and quantified before performing scRNA-seq with Seq-Well v1.

#### Methods to Generate Single-Cell and Bulk RNA-seq Libraries

##### Seq-Well v1

Seq-Well was performed as described (Gierahn et al., 2017). Single cells were diluted to 15,000 cells in 200 μL RPMI + 10% FBS and deposited onto a pre-functionalized PDMS array. 15,000 cells were deposited onto the top of each PDMS array and let settle by gravity into distinct wells. The array was gently washed with PBS, and sealed using a functionalized polycarbonate membrane. Seq-Well arrays were sealed in a dry 37°C oven for 40 min, and submerged in a lysis buffer containing guanidium thiocyanate (Sigma), EDTA, 1% beta-mercaptoethanol and sarkosyl (Sigma) for 20 min at room temperature. Arrays were transferred to hybridization buffer containing NaCl (Fisher Scientific) and agitated for 40 min at room temperature, mRNA capture beads with mRNA hybridized were collected from each Seq-Well array, and beads were resuspended in a master mix for reverse transcription containing Maxima H Minus Reverse Transcriptase and buffer, dNTPs, RNase inhibitor, a 5′ template switch oligonucleotide, and PEG for 30 min at room temperature, and overnight at 52°C with end-over-end rotation. Exonuclease digestion and PCR were carried out as described. Post-whole transcriptome amplification workup involved AMPure XP SPRI bead cleanup occurred at a 0.6 x volume ratio, followed by 0.8x. Library size was analyzed using an Agilent Tapestation hsD5000 kit, confirming the expected peak at ∼1000 bp, and absence of smaller peaks corresponding to primer. Libraries were quantified using Qubit High-Sensitivity DNA kit and prepared for Illumina sequencing using Nextera XT DNA Sample Preparation kit using 900 pg of cDNA library as input to tagmentation reactions. Amplified final libraries were purified twice with AMPure XP SPRI beads as before, with a volume ratio of 0.6x followed by 0.8x. Libraries from 2-3 Seq-Well arrays were pooled and sequenced together using a NextSeq 500/550 High Output v2 kit (75 cycles) using a paired end read structure with custom read 1 primer: read 1: 20 bases, read 2: 50 bases, read 1 index: 8 bases.

##### Seq-Well S^3^

Seq-Well S^3^ modified the following protocol steps from v1, above (Hughes et al., 2019). First, hybridization buffer was supplanted with 8% (v/v) polyethylene glycol (PEG, Sigma). Second, after exonuclease digestion, bead-associated cDNA was denatured for 5 min in 0.2 mM NaOH with end over end rotation. Next, beads were washed with TE + 0.01% tween-20, and second strand synthesis was carried out by resuspending beads in a master mix containing Klenow Fragment (NEB), dNTPs, PEG, and the dN-SMRT oligonucleotide to enable random priming off of the beads.

##### 10X v2 3′

Single cells were loaded onto 3′ library chips as per the manufacturers protocol for Chromium Single Cell 3′ Library (v2) (10X Genomics). Each biopsy was sequenced on two channels of the 10X Chromium Single Cell Platform, one for the epithelial fraction and the other for the lamina propria fraction in order to recover sufficient numbers of epithelial and lamina propria cells for downstream analyses. An input of 6,000 single cells was added to each channel with a recovery rate of approximately 2,000 cells.

##### Drop-seq

Drop-seq experiments were performed according to the original protocol (Macosko et al., 2015). Briefly, single cells (100/μl) were co-encapsulated in droplets with barcoded beads (120/μl, ChemGenes) at rates of 4000 μl/h. Droplet emulsions were collected for 10-20 min/each prior to droplet breakage by perfluorooctanol (Sigma-Aldrich). After breakage, beads were harvested and the hybridized mRNA transcripts reverse transcribed (Maxima RT, Thermo Fisher). Exonuclease digestion and PCR were carried out as described (12 PCR cycles). For each sample, 1 ng of pre-amplified cDNA from an estimated 1000 cells was tagmented by Nextera XT (Illumina) with a custom P5-primer (Integrated DNA Technologies). Single-cell libraries were sequenced in a 100 bp paired-end run on the Illumina HiSeq4000.

##### Smart-Seq2 for Bulk RNA-Seq

Population RNA-seq was performed as described (Ordovas-Montanes et al., 2018, Trombetta et al., 2014). Briefly, RNA from population lysates was purified using AMPure RNA Clean Spri beads (Beckman Coulter) at a 2.2x volume ratio, and mixed with oligo-dT primer, dNTPs (NEB), and RNase inhibitor (Fisher Scientific) at 72°C for 3 min on a thermal cycler to anneal the 3′ primer to polyadenylated mRNA. Reverse transcription was carried out in a master mix of Maxima RNaseH-minus RT enzyme and buffer (Fisher Scientific), MgCl_2_ (Sigma), Betaine (Sigma), RNase inhibitor, and a 5′ template switch oligonucleotide, and PCR was carried out using KAPA HiFi HotStart ReadyMix (Kapa Biosystems) and IS PCR primer and amplified for 18 cycles. Libraries were purified using AMPure XP SPRI beads at a volume ratio of 0.8x followed by 0.9x. Library size was assessed using a High-Sensitivity DNA chip (Agilent Bioanalyzer), confirming the expected size distribution of ∼1,000-2,000 bp. Tagmentation reactions were carried out with the Nextera XT DNA Sample Preparation Kit (Illumina) using 250 pg of cDNA per single cell as input, with modified manufacturer’s instructions as described. Libraries were purified twice with AMPure XP SPRI beads at a volume ratio of 0.9x, size distribution assessed using a High Sensitivity DNA chip (Agilent Bioanalyzer) and Qubit High-Sensitivity DNA kit (Invitrogen). Libraries were pooled and sequenced using NextSeq500/550 High Output v2 kits (75 cycles, Illumina) using 30-30 paired end sequencing with 8-mer dual indexing.

#### Human and Mouse Basal Cell Cytokine Stimulation

Data represented in Figures 5A–5L: Cytokines were added for 12 h overnight at increasing doses (0, 0.1, 0.5, 1, 2, 5, 10 ng/mL) of IL-4 (human: Biolegend 574002), IL-17A (human: Biolegend 570502), IFNγ (human: Biolegend 570202; mouse: Peprotech 315-05), IFNα (human: Biolegend 592702; mouse: Biolegend 752802), or IFNβ (mouse: R&D Systems 8234-MB-010). Each condition was run as a biological triplicate. Data represented in Figure S3C-K: cytokines were added for 12 h overnight at increasing doses (0, 0.1, 0.5, 1, 5, 10 ng/mL) of human IL-4 (Biolegend 574004), IL-13 (Biolegend 571104), IFNα (Biolegend 592704), IFNγ (Biolegend 570204), IL-17A (Biolegend 570504), or IL-1β (Biolegend 579404) (each condition run as a biological quadruplicate). All populations were lysed in 50 μL lysis buffer (RLT + 1% BME, QIAGEN and Sigma, respectively) and snap frozen on dry ice. Bulk RNA-seq was performed as described previously and summarized above (Ordovas-Montanes et al., 2018). Populations were sequenced to an average ± SEM read depth of 3.95 ± 0.11 million reads per sample, with an average ± SEM alignment percentage to either hg19 or mm10 reference transcriptomes of 71 ± 0.3%. All samples met quality thresholds regarding genomic and transcriptomic alignment.

#### Western blot for human ACE2

Established air-liquid interface cultures from bronchial brushings of four asthmatic patients were treated with 10ng/μL of human IFNγ for 24 h. Protein lysates were prepared, and anti-ACE2 human antibody (AF933 R&D goat polyclonal) was used to probe for ACE2 by western blot. Bands were normalized to GAPDH as loading control, and fold change was computed based on normalized ACE2 values.

### Quantification and Statistical Analysis

#### Non-Human Primate Lung and Ileum

Libraries corresponding to 7 animals (variable number of tissues per animal) were sequenced using Illumina NextSeq. Reads were aligned to the *M. mulatta* genome assembly 8.0.1 annotation version 102 and processed according to the Drop-Seq Computational Protocol v2.0 (https://github.com/broadinstitute/Drop-seq). Data was normalized and scaled using the Seurat R package v2.3.4 (https://satijalab.org/seurat/): transforming the data to log_e_(UMI+1) and applying a scale factor of 10,000. To identify major axes of variation within our data, we first examined only highly variable genes across all cells, yielding approximately 1,000-3,000 variable genes with average expression > 0.1 log-normalized UMI across all cells. An approximate principal component analysis was applied to the cells to generate 100 principal components (PCs). Using the JackStraw function within Seurat, we identified significant PCs to be used for subsequent clustering and further dimensionality reduction. For 2D visualization and cell type clustering, we used a Uniform Manifold Approximation and Projection (UMAP) dimensionality reduction technique (https://github.com/lmcinnes/umap) with “min_dist” set to 0.5 and “n_neighbors” set to 30. To identify clusters of transcriptionally similar cells, we employed unsupervised clustering as described above using the FindClusters tool within the Seurat R package with default parameters and k.param set to 10 and resolution set to 0.5. Each cluster was sub-clustered to identify more granular cell types, requiring each cell type to express > 25 significantly upregulated genes by differential expression test (FindMarkers implemented in Seurat, setting “test.use” to “bimod,” Bonferroni-adjusted p value cutoff < 0.001). Differential expression tests between cells from *ACE2*^+^ versus *ACE2*^-^ Type II Pneumocytes were conducted using the SCDE R package with default parameters (Kharchenko et al., 2014). Expression data for epithelial cells and enterocytes included in this dataset can be visualized and downloaded here: https://singlecell.broadinstitute.org/single_cell/study/SCP807?scpbr=the-alexandria-project#study-summary.

#### Human Lung Tissue

Libraries corresponding to 8 donors were sequenced using Illumina NextSeq. Reads were aligned to the hg19 genome assembly and processed according to the Drop-Seq Computational Protocol v2.0 (https://github.com/broadinstitute/Drop-seq). Data was normalized and scaled using the Seurat R package v3.1.0 (https://satijalab.org/seurat/), transforming the data to log_e_(UMI+1) and applying a scale factor of 10,000. For each array, we assessed the quality of constructed libraries by examining the distribution of reads, genes and transcripts per cell. Variable gene selection, principal components analysis, and selection of significant principal components was performed as above. We visualized our results in a two-dimensional space using UMAP (https://github.com/lmcinnes/umap), and annotated each cluster based on the identification of highly expressed genes. To further characterize substructure within cell types (for example, T cells), we performed dimensionality reduction (PCA) and clustering over those cells alone. Sub-clusters (i.e., clusters within broad cell type classifications) were annotated by cross-referencing cluster-defining genes with curated gene lists and online databases SaVanT (http://newpathways.mcdb.ucla.edu/savant-dev/) and GSEA/MsigDB (https://www.gsea-msigdb.org/gsea/msigdb/index.jsp). Proliferating cells from the human lung (Figure 2C) express high levels of mitotic markers, such as *MKI67,* and represent primarily T cells (*CD3D, CD3E*), B cells/antibody-secreting cells (*IGJ, MZB1, IGHG1*), and myeloid cells (*CD14, APOE*) and represent a composite cell cluster. Differential expression analysis between *ACE2+ TMPRSS2+* and negative type II pneumocytes was performed in Seurat using a likelihood-ratio test (FindMarkers implemented in Seurat, setting “test.use” to bimod). Expression data for epithelial cells included in this dataset can be visualized and downloaded here: https://singlecell.broadinstitute.org/single_cell/study/SCP814?scpbr=the-alexandria-project#study-summary.

#### Human Ileum

Libraries corresponding to 13 donors were sequenced using Illumina NovaSeq S2 with a Read 1 26bp, Read 2 91bp, Index 1 8bp configuration before reads were aligned to GRCh38. Each sample was filtered individually for low quality cells and genes by analyzing distributions of reads, transcripts, percent reads mapped to mitochondrial genes, and complexity per cell, then merged as an outer join to create a single dataset. Clustering and differential expression tests were processed using Seurat v3.1.0 (https://satijalab.org/seurat/). Normalization and variable gene selection was processed with SCTransform (https://github.com/ChristophH/sctransform). Clustering for major cell types was performed using Louvain clustering on dimensionally reduced PCA space with resolution set via grid search optimizing for maximum average silhouette score. Due to the scale of the dataset, a randomized subsampling from across the dataset was used to calculate the silhouette score. We annotated clusters based on highly expressed genes, then sub-clusters were characterized by performing PCA dimensionality reduction and clustering over those cells alone, and annotated based on highly expressed genes found via one-versus-rest differential expression test (Wilcoxon) within the major cell type. Differential expression analysis between *ACE2*^+^*TMPRSS2*^+^ and negative epithelial cells was performed in Seurat using a Wilcoxon test and Bonferroni p value correction. Expression data for epithelial cells included in this dataset can be visualized and downloaded here: https://singlecell.broadinstitute.org/single_cell/study/SCP812?scpbr=the-alexandria-project#study-summary.

#### Human Adult Nasal Mucosa

Sample processing, sequencing, and analysis was performed as in (Ordovas-Montanes et al., 2018). Briefly, scRNA-seq cell suspensions were freshly processed using Seq-Well v1 and Seurat v2.3.4 was utilized for computational analyses presented here (Butler et al., 2018, Satija et al., 2015). Cell by gene matrix and R code for initialization of object available to download as Supplemental Data and Supplementary Tables here https://www.nature.com/articles/s41586-018-0449-8 and here:

http://shaleklab.com/resource/mapping-allergic-inflammation/? and visualized here: https://singlecell.broadinstitute.org/single_cell/study/SCP253?scpbr=the-alexandria-project#study-summary. Scores for various cytokines acting on human airway epithelial cells were calculated based on gene lists derived for (Ordovas-Montanes et al., 2018), calculated using AddModuleScore function Seurat, and effect size calculated by Cohen’s d, as previously reported.

#### Granulomatous Tissue from Mycobacterium Tuberculosis Infected NHPs

Libraries corresponding to 10 animals (variable number of tissues/animal) were sequenced using Illumina NovaSeq S2. Data was aligned using the Dropseq-tools pipeline on Terra (app.terra.bio) to *M. fascicularis* reference genome assembly 5, annotation version 101. Clustering was performed using Leiden clustering in the Scanpy (scanpy.readthedocs.io) package (Wolf et al., 2018). Cell type labels were assigned using known marker genes. In this analysis, we include all epithelial cell subsets (secretory, multiciliated, type II pneumocytes, and type I pneumocytes) from all samples. Differential expression between *ACE2*^+^*TMPRSS2*^+^ cells and other cells of the matched cell subtype (e.g., Secretory Cells) were performed using the “bimod” likelihood-ratio test within each cell subtype and filtered on Benjamini-Hochberg-corrected p value < 0.05. Expression data for epithelial cells included in this dataset can be visualized and downloaded here:

https://singlecell.broadinstitute.org/single_cell/study/SCP806?scpbr=the-alexandria-project#study-summary.

#### Basal Cell Cytokine Stimulation

Libraries corresponding to 279 populations were sequenced using Illumina NextSeq. Reads were aligned to the hg19 or mm10 genome assembly using the cumulus platform https://cumulus-doc.readthedocs.io/en/0.12.0/smart_seq_2.html and output as TPM using RSEM v1.3.2. Populations were transformed to transcripts per 10K reads and log2(1+TP10K) transformed. *ACE2* expression by stimulation condition and dose were assessed using one-way ANOVA with post hoc testing using a Bonferroni correction. Plots were generated using ggplot2, and transcriptome-wide differential expression was calculated using the Seurat R package v3.1.0 (https://satijalab.org/seurat), function FindMarkers with test.use = ”bimod.” Expression data can be visualized and downloaded here:

https://singlecell.broadinstitute.org/single_cell/study/SCP822?scpbr=the-alexandria-project.

#### Interferon Treatment of Mouse Nasal Mucosa

Libraries corresponding to 4 mice, with 2 Seq-Well arrays per mouse were sequenced using Illumina NextSeq as described (Gierahn et al., 2017, Hughes et al., 2019). Reads were aligned to the mm10 genome and processed according to the Drop-Seq Computational Protocol v2.0 (https://github.com/broadinstitute/Drop-seq). Data was normalized and scaled using the Seurat R package v2.3.4 (https://satijalab.org/seurat/): transforming the data to log_e_(UMI+1) and applying a scale factor of 10,000. Cells with fewer than 1000 UMIs and 500 unique genes were removed. To identify major axes of variation within our data, we first examined only highly variable genes across all cells, yielding approximately 5,000 variable genes. An approximate principal component analysis was applied to the cells to generate 200 principal components (PCs). Using a combination of the Jackstraw function in Seurat and observing the “elbow” of the standard deviations of PCs, we chose the top 70 PCs for subsequent clustering and visualization. For 2D visualization, we used a Uniform Manifold Approximation and Projection (UMAP) dimensionality reduction technique (https://github.com/lmcinnes/umap) with “min_dist” set to 0.3 and “n_neighbors” set to 50. To identify clusters of transcriptionally similar cells, we employed unsupervised clustering as described above using the FindClusters tool within the Seurat R package with default parameters and k.param set to 10. Resolution was chosen based on maximization of the average silhouette width across all cells. Clusters were merged if a cell type expressed fewer than 25 significantly upregulated genes by differential expression test (FindAllMarkers implemented in Seurat, setting “test.use” to “bimod,” Bonferroni-adjusted p value cutoff < 0.001). Differential expression tests between cells from saline-treated or IFNa-treated mice were assessed using the FindMarkers function with “test.use” set to “bimod. This dataset can be visualized and downloaded here:

https://singlecell.broadinstitute.org/single_cell/study/SCP832?scpbr=the-alexandria-project#study-summary.

#### Lung from MHV68-Infected WT and IFNγR KO Mice

Libraries corresponding to 14 mice were aligned to a custom reference genome encompassing both murine (mm10) and herpes virus genes: 84 known genes from MHV68 were retrieved from NCBI (NCBI: txid33708) and added to the mm10 mouse genome. Reads were aligned to the custom joint genome and processed according to the Drop-Seq Computational Protocol v2.0 (https://github.com/broadinstitute/Drop-seq). Barcodes with < 200 unique genes, > 20,000 UMI counts, and > 30% of transcript counts derived from mitochondrially encoded genes were discarded. Data analysis was performed using the Scanpy Package following the common procedure, the expression matrices were normalized using *scran*’s size factor based approach and log transformed via scanpy’s pp.log1p() function (Lun et al., 2016, Wolf et al., 2018). SoupX was utilized to reduce ambient RNA bias, using default parameters with pCut set to 0.3, and was applied to each sample before merging the count matrices (Young and Behjati, 2020). UMI per cell and cell cycle were regressed out. Highly variable genes were selected by running pp.highly_variable_genes() for each sample separately, returning the top 4,000 variable genes per sample, and genes identified in variable in > 5 samples were retained, yielding 14,305 genes. Next, only *Epcam*+ cells were considered, principal components (PCs) were calculated using only the selected variable genes, and 6 PCs were used to perform unsupervised Louvain clustering. Type I Pneumocytes were excluded from this analysis based on uniformly negative expression of *Ace2*, resulting in a final dataset subset of 5,558 cells. Cells were identified as infected if at least one viral read was detected.

#### Nasal Washes during Influenza Infection

Sample processing, sequencing, and analysis was performed as in (Cao et al., 2020). Reads were aligned to the GRCh37 reference genome combined with influenza genomes. Mapped reads from each sample were then corrected for Drop-seq barcode synthesis error using the Drop-seq core computational tools developed by the McCarroll Lab (Macosko et al., 2015). Genes were quantified using End Sequence Analysis Toolkit (ESAT, github/garber-lab/ESAT) with parameters *-wlen 100 -wOlap 50 -wExt 0 -scPrep* (Derr et al., 2016). Finally, UMIs that likely result from sequencing errors were corrected by merging any UMIs that were observed only once and have 1 hamming distance from a UMI detected by two or more aligned reads. Only cell barcodes with more than 1,000 UMIs were analyzed. Cell barcodes with mostly erythrocyte genes (*HBA, HBB*) were removed. From here on, the remaining cell barcodes in the matrix would be referred to as cells. The final gene by cell matrix was normalized using the scran package v3.10 (Lun et al., 2016). The normalized matrix was used for dimensionality reduction by first selecting variable genes that had a high coefficient of variance (CV) and were expressed (> = 1 UMI) by more than three cells. Influenza viral genes, interferon stimulated genes, and cell cycle related genes were removed from the variable gene list in order to minimize the impact of viral responses and mitosis on clustering and cell type identification. This resulted in the selection of 2484 variable genes. t-distributed stochastic neighbor embedding (tSNE) was applied to the first ten principal components (PCs), which explained 95% of the total data variance. Density clustering (Rodriguez and Laio, 2014) was performed on the resulting tSNE coordinates and identified four major clusters: epithelial cells, neutrophils, macrophages and leukocytes. The epithelial cell cluster and the leukocyte cluster were then re-clustered independently, as described above, to identify populations within each metacluster. Specifically, the epithelial cell cluster was re-embedded using 2629 variable genes selected by the same criteria mentioned in the previous section and 13 PCs that explained 95% of the variance. Density clustering over the epithelial cell subset revealed ten clusters. Differential gene expression analysis using edgeR (Robinson et al., 2010) was performed to identify marker genes for each cluster. Influenza-infected and bystander cells were identified after correcting for sample-specific distribution of ambient influenza mRNA contamination and predicted cells most likely to be infected identified using a hurdle zero inflated negative binomial (ZINB) model and a support vector machine (SVM) classifier.

#### Power Calculations for Detection of Rare Transcripts

We conducted the following statistical analysis to estimate the effects of various factors on our ability to make confident claims regarding the presence/absence of transcripts of interest (e.g., *ACE2*), both within individual cells and clusters (Figure S6). Specifically, we investigated the roles of capture/reverse transcription efficiency, *ACE2* expression level, sequencing depth, and cell numbers. Taken together, the results of this power analysis are in agreement with other efforts to model biological and technical sources of zero-inflation within scRNA-seq data (e.g., https://satijalab.org/howmanycells and Kharchenko et al., 2014, Svensson, 2020).

We began by quantifying how likely we are to capture and transcribe at least one *ACE2* mRNA molecule, as a function of the number *ACE2* mRNA molecules per cell and a protocol’s efficiency (Figure S6A). Drop-Seq has a capture/transcription efficiency of ∼10% (as estimated using ERCC spike ins; see (Macosko et al., 2015), and the experimental platforms used in this study are either equivalent (e.g., Seq-Well v1, (Gierahn et al., 2017) or superior (e.g., 10-fold better unique molecule detection, 5-fold better gene detection using Seq-Well S^3^;(Hughes et al., 2019)). Most relevant to this context, inferior turbinate scrapings were processed using both Seq-Well v1 and Seq-Well S^3^ (Figure S3B). Importantly, Seq-Well S^3^ provided > two-fold increase in the detection frequency of rare *ACE2* transcripts (i.e., *ACE2*+: 4.7% for v1 versus 9.8% for S^3^), making it reasonable to expect that such improvements in single-cell experimental technologies have yielded corresponding improvements in capture and transcription efficiency. Based on Drop-Seq’s 10% efficiency, even if *ACE2* is expressed at the low level of 5 mRNA molecules per cell (a reasonable order-of-magnitude estimate, given that non-human primate ileum cells had a maximum of 10 *ACE2* unique molecules per cell observed via sequencing and an average of 1.93 molecules per cell in expressing cells, see Figures 3B and 3C), our experimental platforms have a minimum likelihood of 41% to capture and reverse transcribe at least one *ACE2* mRNA molecule in any given individual cell. This likelihood rapidly increases if we estimate higher efficiencies for improved scRNA-seq technologies (e.g., 67% likelihood within any individual cell at 20% capture/transcription efficiency, 76% likelihood at 25% efficiency, Figure S6A). Thus, while transcript drop-out may reduce the fraction of positive cells, with the capture and transcription efficiencies of improved single-cell technologies, the impact is likely to be minor (reads are likely underestimated by up to a factor of ∼2.5x), given a sufficient depth of sequencing (see below). We note that this impacts both clusters deemed to contain and not contain *ACE2*+ cells, and suggests our percentages are likely lower bounds for true expression (within a factor of ∼2.5x).

Next, we examined the probability of sequencing an *ACE2* transcript as a function of read depth and *ACE2*’s fractional abundance in each single cell within our sequencing libraries. First, across two different tissues (non-human primate ileum and lung, representing a high expresser of *ACE2* and low expresser, respectively), we calculated the proportion of unique *ACE2* molecules in our *ACE2*+ cells (defined as any cell with at least 1 UMI aligning to *ACE2*) as a fraction of total reads within individual cells to provide an order-of-magnitude estimate for average *ACE2* abundance in our single-cell sequencing libraries (i.e., the probability that a read within a cell corresponds to a unique molecule of *ACE2*, Figure S6B). We highlight that by calculating probabilities based on *ACE2* unique molecules divided by an individual cell’s total reads, we are providing a conservative estimate for the probability of observing *ACE2* as a function of sequencing depth (e.g., as compared to basing these probabilities on *ACE2* non-UMI-collapsed reads divided by total reads). Next, we obtained information on the number of reads in these cell populations to provide estimates of average sequencing depths (Figure S6C). Using the mean fractional abundances of ACE2 from each tissue (Figure S6B) and the mean read depths for all genes (Figure S6C), we calculated the probability of detecting at least 1 *ACE2* molecule (i.e., P(detecting > 0 ACE2 molecules) = 1 - (1 - ACE2 fractional abundance)^Read depth^). This results in a 93.7% probability in ileum-derived cell libraries that contain *ACE2*, and a 76.0% probability for lung-derived cell libraries, indicating that our sequencing depths are sufficient to detect *ACE2*+ cells (Figure S6D).

To further evaluate whether our ability to detect *ACE2*+ cells was an artifact of sequencing depth, we compared the number of *ACE2*+ cells in a cluster to the mean number of reads across all cells in that same cluster (Figure S6E). We did not observe any significant correlation: the ileum cell cluster with the highest number of *ACE2*+ cells had the lowest sequencing depth of all ileum clusters, and the lung cell cluster with the highest number of *ACE2*+ cells was approximately average in its read depth (on a log-log scale, Pearson’s r = −0.31, non-significant). Further, when comparing *ACE2*+ cells to *ACE2*- cells within a given tissue, we did not observe a positive correlation between read depth and *ACE2* status (i.e., mean ± standard error of the mean, SEM, reads among all lung cells = 28,512 ± 344; mean ± SEM reads among *ACE2*+ lung cells = 28,553 ± 2,988; mean ± SEM reads among all ileum cells = 14,864 ± 288; mean ± SEM reads among *ACE2*+ ileum cells = 10,591 ± 441, full statistics on cell depth among *ACE2*+ cells compared to *ACE2*- cells of the same cell type can be found in Table S9). Thus, we can be confident that the observed differences in *ACE2*+ proportions across clusters are not driven by differences in sequencing depth.

Finally, we investigated how observed differences in *ACE2*+ proportions across clusters might be affected by cell sampling. Using the proportion of *ACE2*+ cells in a “typical” cluster annotated as being *ACE2* positive (i.e., 6.8% in non-human primate type II pneumocytes, Figure 1), we calculated the cluster sizes needed to be confident that the probability of observing zero to a few positive cells is unlikely to have arisen by random chance (probabilities calculated under a negative binomial distribution with parameter p = 0.068, Figure S6E). We found that as cluster sizes approach and exceed 100 cells, the probability of observing zero to a few positive cells rapidly approaches zero, if we assume 6.8% of cells are positive. Further, to examine our confidence in estimating an approximate upper bound (ignoring the impact of protocol inefficiencies discussed above) for the fraction of cells positive in a cluster as a function of the number of cells in that cluster, we also calculated the probability of observing zero (and its complement, probability of observing at least 1) *ACE2*+ cells as a function of cluster size across true positive proportions ranging from 0.1% to 10% (probabilities calculated under a negative binomial distribution with parameter p = 0.001 to 0.1, representing hypothetical proportions of *ACE2*+ cells Figure S6F). Given our typical cluster sizes (on the order of hundreds of cells, exact values provided in Table S9), we find that for us to observe 0 *ACE2*+ cells in a cluster due to sampling artifacts, the fraction of true positives must be ∼1% or less. Thus, these complementary approaches demonstrate that our observed variations in *ACE2*+ cell proportions across clusters likely reflect underlying biological differences, rather than random chance.

#### Statistical Testing

Parameters such as sample size, number of replicates, number of independent experiments, measures of center, dispersion, and precision (mean ± SEM) and statistical significances are reported in Figures and Figure Legends. A p value less than 0.05 was considered significant. Where appropriate, a Bonferroni or FDR correction was used to account for multiple tests, alternative correction methods are noted in the figure legends or Methods. All statistical tests corresponding to differential gene expression are described above and completed using R language for Statistical Computing.

## Consortia

The members of HCA Lung Biological Network are Nicholas E. Banovich, Pascal Barbry, Alvis Brazma, Tushar Desai, Thu Elizabeth Duong, Oliver Eickelberg, Christine Falk, Michael Farzan, Ian Glass, Muzlifah Haniffa, Peter Horvath, Deborah Hung, Naftali Kaminski, Mark Krasnow, Jonathan A. Kropski, Malte Kuhnemund, Robert Lafyatis, Haeock Lee, Sylvie Leroy, Sten Linnarson, Joakim Lundeberg, Kerstin B. Meyer, Alexander Misharin, Martijn Nawijn, Marko Z. Nikolic, Jose Ordovas-Montanes, Dana Pe’er, Joseph Powell, Stephen Quake, Jay Rajagopal, Purushothama Rao Tata, Emma L. Rawlins, Aviv Regev, Paul A. Reyfman, Mauricio Rojas, Orit Rosen, Kourosh Saeb-Parsy, Christos Samakovlis, Herbert Schiller, Joachim L. Schultze, Max A. Seibold, Alex K. Shalek, Douglas Shepherd, Jason Spence, Avrum Spira, Xin Sun, Sarah Teichmann, Fabian Theis, Alexander Tsankov, Maarten van den Berge, Michael von Papen, Jeffrey Whitsett, Ramnik Xavier, Yan Xu, Laure-Emmanuelle Zaragosi, and Kun Zhang. Pascal Barbry, Alexander Misharin, Martijn Nawijn, and Jay Rajagopal serve as the coordinators.

## Supplemental Information

Document S1. Author contributions

Table S1 Statistics and Gene Lists Related to Figure 1 (Non-Human Primate Lung) A. Cluster defining genes for cell types presented in Figure 1. B. *ACE2* and *TMPRSS2* statistics by cell type. C. Differentially expressed genes between *ACE2*^+^ and *ACE2*^-^ Type II Pneumocytes.

Table S2 Statistics and Gene Lists Related to Figure 2 (Human Lung) A. Cluster defining genes for cell types presented in Figure 2. B. *ACE2* and *TMPRSS2* statistics by cell type. C. Differentially expressed genes between *ACE2*^+^*TMPRSS2*^+^ versus rest Type II Pneumocytes.

Table S3 Statistics and Gene Lists Related to STAR Methods and Figure S1 (NHP Tuberculosis Infected Lung and Granuloma) A. Cluster defining genes for cell types presented in Figure S1 B. *ACE2* and *TMPRSS2* statistics by cell type C. Abundances of *ACE2*^+^*TMPRSS2*^+^ cells by cell type and tissue of origin D. Differentially expressed genes between *ACE2*^+^*TMPRSS2*^+^ versus rest Type II Pneumocytes

Table S4 Statistics and Gene Lists Related to Figure 3 (Non-Human Primate Ileum) A. Cluster defining genes for cell types presented in Figure 3B B. *ACE2* and *TMPRSS2* statistics by cell type. C. Differentially expressed genes between *ACE2*^+^*TMPRSS2*^+^ versus rest Absorptive Enterocytes

Table S5 Statistics and Gene Lists Related to Figure 3 (Human Ileum) A. Cluster defining genes for cell types presented in Figure 3D B. *ACE2* and *TMPRSS2* statistics by cell type. C. Differentially expressed genes between *ACE2*^+^*TMPRSS2*^+^ Absorptive Enterocytes versus rest Enterocytes D. Differentially expressed genes between *ACE2*^+^*TMPRSS2*^+^ Absorptive Enterocytes versus rest Absorptive Enterocytes

Table S6 Statistics and Gene Lists Related to Figure 4 and 5 (Human Nasal Mucosa and Human Influenza) A. *ACE2* and *TMPRSS2* statistics by cell type presented in Figure 4B. B. Cluster defining genes for cell types presented in Figure 4C and 4D. C. Cluster defining genes for cell types presented in Figure 4C and 4F. D. *ACE2* and *TMPRSS2* statistics by cell type presented in Figure 4C. E. *ACE2* and *TMPRSS2* statistics by cell type presented in Figure 4D. F. Differentially expressed genes between *ACE2*^+^*TMPRSS2*^+^ epithelial cells versus rest epithelial cells in Figure 4E G. Differentially expressed genes between *ACE2*^+^*TMPRSS2*^+^ epithelial cells versus rest secretory epithelial cells in Figure 4E H. Raw data for cell type abundances by tissue and disease, Figure 4H I. Differentially expressed genes between *ACE2*^+^*TMPRSS2*^+^ versus Goblet Cells from Human Influenza dataset (Figure 6E-G).

Table S7 Differentially Expressed Genes in Human and Mouse Basal Epithelial Cells Treated with Various Cytokines and Interferons, Related to Figure 5 A. Mouse Untreated versus IFNβ B. Mouse Untreated versus IFNα2 C. Mouse Untreated versus IFNγ D. BEAS-2B Untreated versus IL4 E. BEAS-2B Untreated versus IL17A F. BEAS-2B Untreated versus IFNγ G. BEAS-2B Untreated versus IFNα2 H. Human Donor 1 Untreated versus IL4 I. Human Donor 1 Untreated versus IL17A J. Human Donor 1 Untreated versus IFNγ K. Human Donor 1 Untreated versus IFNα2 L. Human Donor 2 Untreated versus IL4 M. Human Donor 2 Untreated versus IL17A N. Human Donor 2 Untreated versus IFNγ O. Human Donor 2 Untreated versus IFNα2

Table S8 Statistics and Gene Lists Related to Figure 6 and Figure S5 (Mouse Nasal Epithelium) A. Cluster defining genes for cell types presented in Figure 6A B. *Ace2* and *Tmprss2* statistics by cell type C. Differentially expressed genes among Basal Epithelial Cells treated with Saline versus Intranasal IFNα

Table S9 Summary of Datasets Analyzed in this Study with Links to Data, Related to STAR Methods
